# Crosslinking reactions of 4-amino-6-oxo-2-vinylpyrimidine with guanine derivatives and structural analysis of the adducts

**DOI:** 10.1093/nar/gkv797

**Published:** 2015-08-05

**Authors:** Shuhei Kusano, Shogo Ishiyama, Sik Lok Lam, Tsukasa Mashima, Masato Katahira, Kengo Miyamoto, Misako Aida, Fumi Nagatsugi

**Affiliations:** 1 Institute of Multidisciplinary Research for Advanced Materials, Tohoku University, 2-1-1 Katahira, Aoba-ku, Sendai-shi, Miyagi 980-8577, Japan; 2 Department of Chemistry, The Chinese University of Hong Kong, Shatin, New Territories, Hong Kong, China; 3 Institute of Advanced Energy, Graduate School of Energy Science, Kyoto University, Gokasho, Uji, Kyoto 611-0011, Japan; 4 Department of Chemistry, Graduate School of Science, Hiroshima University,1-3-1, Kagamiyama, Higashi-Hiroshima, Hiroshima 739-8526, Japan

## Abstract

DNA interstrand crosslinks (ICLs) are the primary mechanism for the cytotoxic activity of many clinical anticancer drugs, and numerous strategies for forming ICLs have been developed. One such method is using crosslink-forming oligonucleotides (CFOs). In this study, we designed a 4-amino-6-oxo-2-vinylpyrimidine (AOVP) derivative with an acyclic spacer to react selectively with guanine. The AOVP CFO exhibited selective crosslinking reactivity with guanine and thymine in DNA, and with guanine in RNA. These crosslinking reactions with guanine were accelerated in the presence of CoCl_2_, NiCl_2_, ZnCl_2_ and MnCl_2_. In addition, we demonstrated that the AOVP CFO was reactive toward 8-oxoguanine opposite AOVP in the duplex DNA. The structural analysis of each guanine and 8-oxoguanine adduct in the duplex DNA was investigated by high-resolution NMR. The results suggested that AOVP reacts at the N2 amine in guanine and at the N1 or N2 amines in 8-oxoguanine in the duplex DNA. This study demonstrated the first direct determination of the adduct structure in duplex DNA without enzyme digestion.

## INTRODUCTION

**Figure 1.**
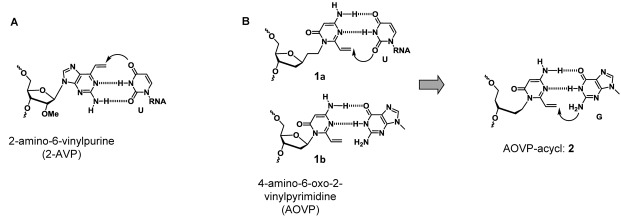
**(A)** Selective crosslinking reactions to uracil (U) in RNA with 2-AVP. **(B)** Molecular design for the acyclic pyrimidine.

Strategies for preparing crosslinked duplex DNA have attracted attention due to their many applications in a variety of fields, including DNA repair, gene regulation and nanotechnology. DNA interstrand crosslinks (ICLs) are the primary mechanism for the cytotoxic activity of many clinical anticancer drugs, such as nitrogen mustards and platinum agents (1,2). Drug resistance in tumor cells through enhanced ICL repair is a major problem in cancer treatment (3,4). Although a number of repair pathways have been implicated in ICL repair, the molecular mechanism remains poorly understood (5,6). Determining the chemical structure of crosslinked duplex DNA could help elucidate the repair mechanism (7). Covalently linked duplex DNA can be prepared by using a variety of crosslinked dinucleotides (8–15). Oligonucleotides (ODNs) containing O^6^-guanine-alkyl-O^6^-guanine ICL products were used to investigate the repair of DNA ICLs by O^6^-alkylguanine-DNA alkyltransferase (16,17). Plasmids containing N^4^C-ethylN^4^C that mimicked nitrogen mustard ICL, and N^3^T-ethyl-N^3^T or N^1^I-ethyl-N^3^T ICL that mimicked the nitrosourea ICL structure were used to investigate the repair mechanism in cells (18). In an alternative approach, duplex DNA that contained a reactive moiety in both strands was used to prepare covalently linked duplex DNA (19–27). ICL duplex DNA has been synthesized by disulfide bond linkage (21,27), click chemistry (25,26) and amide bond formation (22). These strategies produced a variety of ICL duplex DNA structures by adjusting the linker length between the DNA strand and each reactive moiety and these strategies were used to form the DNA nanostructure. However, these methods for preparing ICL duplex DNA could not be used to control gene regulation. Crosslink-forming oligonuleotides (CFOs) bind to the target mRNA to form an irreversible complex, and effectively inhibit translation. Various functional groups have been developed for ICL formation (28) by photoirradiation, including psoralen (29,30), diaziridine (31) and carbazoles (32). In addition, reactive functional groups activated by a chemical reaction have been reported, such as quinone methides (33,34), furan derivatives (35,36) and modified pyrimidine derivatives (37,38). For the other reactive moiety for the ICL reactions, we developed 2-amino-6-vinylpurine (2-AVP) (Figure 1A). The 2′-OMe RNA containing 2-AVP selectively forms a covalent linkage with the complementary sequence of mRNA at the uridine residue across the AVP (39). The high selectivity and reactivity of this CFO could be attributed to the close proximity of the vinyl group of 2-AVP to uridine in the hybridized complex. The 2-AVP CFO can bind to the mRNA and suppress translation *in vitro*, which leads to the production of the truncated protein (40). In addition, our CFO can inhibit the miRNA function by in-cell crosslinking to the mRNA (41).

Based on the structure of 2-AVP, we also designed 4-amino-6-oxo-2-vinylpyrimidine (AOVP) (Figure 1B). We demonstrated that the nucleoside derivative (**1a**) with an ethyl spacer between the sugar moiety and the reactive base exhibited a fast, selective ICL reaction with thymine (42). In this study, we designed an acyclic AOVP derivative (**2**) expected to crosslink with guanine. The distance between the reactive base and sugar moiety of **2** is shorter than that of **1**, and **2** could also form two hydrogen bonds with guanine. Thus, the acyclic AOVP (**2**) was forced into the proximity to guanine and expected the efficient crosslinking reaction with guanine. Moreover, **2** is a ribose ring-opened analogue of **1b**, which was previously designed to react with guanine residue. However, the synthesis of **1b** failed because the glycosylation of AOVP with the sugar moiety was difficult. In this work, we synthesized an ODN containing **2** and evaluated the crosslinking reactivity. Furthermore, we analyzed the structure of the crosslinked products by high-resolution NMR.

## MATERIAL AND METHODS

### Synthesis of the AOVP derivative phosphoramidite

#### 4-amino-2-(2-octylthioethyl)-6-oxo-1-(3′,5′-*O*-dibenzyl-2′,4′-dideoxy-D-ribityl)pyrimidine (5)

Lithium hydride (109 mg, 13.7 mmol) was added to a suspension of **3** (532 mg, 1.88 mmol) and **4** (995 mg, 2.53 mmol) in dioxane (19 ml). After stirring for 30 min at room temperature, the reaction mixture was heated to 100ºC and stirred for 5 days. After cooling to room temperature, saturated NH_4_Cl was added to the reaction mixture. The layers were separated and the aqueous phase was extracted with CH_2_Cl_2_. The organic phase was washed with brine, dried over anhydrous Na_2_SO_4_, filtered, and concentrated in *vacuo* to obtain an oil. The residue was purified by column chromatography (CHCl_3_/MeOH, 1:0 to 40:1) to afford **5** (366 mg, 34%) as a pale yellow oil; ^1^H NMR (400 MHz, CDCl_3_) δ 0.879 (t, *J* = 6.8 Hz, 3H), 1.26–1.34 (m, 10H), 1.55 (quint, *J* = 8.0 Hz, 2H), 1.77–2.02 (m, 4H), 2.48 (t, *J* = 8.0 Hz, 2H), 2.84–2.91 (m, 4H), 3.58 (t, *J* = 2.8 Hz, 2H), 3.73–3.76 (m, 1H), 3.90–3.97 (m, 1H), 4.04–4.44 (m, 1H), 4.48 (d, *J* = 4.0 Hz, 2H), 4.54 (d, *J* = 2.8, 2H), 4.62 (brs, 2H), 7.27–7.36 (m, 10H); ^13^C NMR (100 MHz, CDCl_3_) δ 14.1, 22.6, 28.7, 28.8, 29.2, 29.6, 31.8, 32.5, 33.3, 34.0, 34.6, 39.7, 66.5, 71.2, 73.0, 74.1, 86.0, 127.5, 127.6, 127.7, 127.9, 128.3, 128.4, 138.3, 138.4, 160.0, 160.9, 163.1; HRMS-ESI (*m/z*): [M+Na]^+^ calcd for C_33_H_47_N_3_NaO_3_S, 588.3230; found, 588.3229.

#### 2-(2-octylthioethyl)-6-oxo-4-phenoxyacetylamino-1-(3′,5′-*O*-dibenzyl-2′,4′-dideoxy-D-ribityl) pyrimidine (6)

Phenoxyacetyl chloride (0.18 ml, 1.31 mmol) was added to a solution of **11** (366 mg, 0.647 mmol) in pyridine (6.5 ml) at 0ºC. After stirring for 1h at 0ºC, the reaction mixture was allowed to warm to room temperature. After additional stirring at room temperature for 4h, the reaction mixture was diluted with CH_2_Cl_2_, washed with saturated NaHCO_3_ and brine, dried over anhydrous Na_2_SO_4_, filtered, and concentrated in *vacuo*. The residue was purified by column chromatography (hexane/ethyl acetate, 5:1 to 2:1) to afford **12** (282 mg, 62%) as a pale yellow oil; ^1^H NMR (400 MHz, CDCl_3_) δ 0.874 (t, *J* = 6.8 Hz, 3H), 1.26–1.35 (m, 11H), 1.56 (quint, *J* = 7.6 Hz), 1.76–2.03 (m. 10H), 2.50 (t, *J* = 7.6 Hz, 2H), 2.86 (t, *J* = 6.8 Hz, 2H), 2.95 (t, *J* = 6.8 Hz, 2H), 3.56 (t, *J* = 5.6 Hz, 2H), 3.73–3.79 (m, 1H), 3.95–4.02 (m, 1H), 4.08–4.15 (m, 1H), 4.46 (d, *J* = 2.0 Hz, 2H), 4.61 (s, 2H); 6.98 (d, *J* = 8.8 Hz, 2H), 7.07 (t, *J* = 8.0 Hz, 1H), 7.18 (s, 1H), 7.21–7.37 (m, 12H), 8.44 (s, 1H); ^13^C NMR (100 MHz, CDCl_3_) δ 14.1, 22.6, 28.6, 28.8, 29.1, 29.5, 31.8, 32.5, 32.9, 34.0, 34.5, 40.4, 66.5, 67.4, 71.2, 73.1, 74.1, 97.2, 114.8, 122.6, 127.5, 127.6, 127.7, 127.8, 127.8, 128.3, 128.4, 129.9, 138.2, 138.3, 152.4, 156.8, 160.2, 163.2, 167.0; HRMS-ESI (*m/z*): [M+Na]^+^ calcd for C_41_H_53_N_3_NaO_3_S, 722.3598; found, 722.3596.

#### 2-(2-octylthioethyl)-6-oxo-4-phenoxyacetylamino-1-(5′-*O*-(4,4′- dimethoxytrityl)-2′,4′-dideoxy-D-ribityl)pyrimidine (8)

Pd(OH)_2_/C was added to a solution of **6** (509 mg, 0.727 mmol) in MeOH (22 ml) under an argon atmosphere. The argon in the reaction flask was evacuated and reaplaced with H_2_. After stirring for 17 h at room temperature, the solution was filtered through a pad of Celite^®^. The solution was then concentrated in *vacuo*. The residue was purified by column chromatography (CHCl_3_-MeOH, 1:0 to 45:1) to afford **7** (212 mg, 56%) as a colorless oil; ^1^H NMR (400 MHz, CDCl_3_) δ 0.876 (t, *J* = 6.8 Hz, 3H), 1.26–1.35 (m, 10H), 1.59–1.83 (m, 6H), 1.76–2.03 (m. 4H), 2.57 (t, *J* = 8.0 Hz, 2H), 2.94–3.08 (m. 5H), 3.71–3.89 (m. 4H), 4.51–4.61 (m. 2H), 7.00 (d, *J* = 7.6 Hz, 2H), 7.08 (t, *J* = 7.6 Hz, 1H), 7.27 (s, 1H), 7.36 (t, *J* = 7.6 Hz, 1H), 8.44 (s, 1H); ^13^C NMR (100 MHz, CDCl_3_) δ 14.1, 22.6, 28.7, 28.8, 29.2, 29.6, 31.8, 32.7, 34.4, 37.1, 38.0, 61.6, 67.5, 67.6, 97.0, HRMS-ESI (*m/z*): [M+Na]^+^ calcd for C_27_H_41_N_3_NaO_3_S, 542.2659; found, 542.2657.

#### 2-(2-octylthioethyl)-6-oxo-4-phenoxyacetylamino-1-(3′-*N*,*N*-diisopropylcyanoethylphosphor amidyl-5′-*O*-(4,4′-dimethoxytrityl)-2′,4′-dideoxy-D-ribityl)pyrimidine (9)

2-cyanoethyl *N*,*N*-diisopropylchlorophosphoramidite (43.6 μl, 199 mmol) was added to a solution of **8** (54.5 mg, 66.3 μmol) and DIPEA (65.0 μl, 0.398 mmol) in CH_2_Cl_2_ (1.3 ml) at 0ºC. After stirring for 1 h at 0ºC, the reaction mixture was diluted with CH_2_Cl_2_, washed with sat. NaHCO_3_ and brine, dried over anhydrous Na_2_SO_4_, filtered and concentrated in *vacuo*. The residue was then purified by column chromatography (hexane-ethyl acetate, 2:1 to 1:1, 1% Et_3_N) to afford **9** (53.4 mg, 79%) as a white foam; ^31^P NMR (162 MHz, CDCl_3_).

### Synthesis of oligonucleotides containing AOVP

ODN**1** was synthesized on a 1 μmol scale with an ABI 392 DNA/RNA synthesizer by standard β-cyanoethyl phosphoramidite chemistry. 5′-Terminal dimethoxytrityl-bearing ODN**1** was removed from the solid support by treatment with 28% NH_3_ (0.5 ml) and the residue was evaporated under reduced pressure. The crude product was purified by reverse-phase HPLC using a C-18 column (COSMOSIL 5C18-MS-II, Nacalai Tesque: 10 × 250 mm) by a linear gradient of 10–40%/20 min of acetonitrile in 0.1 M TEAA buffer at a flow rate of 4 ml/min. The dimethoxytrityl group of the purified ODN was removed with 10% AcOH and the mixture was purified by ethanol precipitation to obtain ODN**1**.

MALDI-TOF MS (*m*/*z*) **ODN1**: [M-H]^−^ calcd. 4422.1; found 4421.0.

To a solution of **ODN1** (122 μl, 15.0 nmol) was added a solution of magnesium bis(monoperoxy phtalate)hexahdrate (MMPP) (15 μl, 75.0 nmol) in carbonate buffer adjusted to pH 10 at room temperature. After 1 h, NaOH (4 M, 10.5 μl) was added, and the mixture was left for an additional 30 min to form the **ODN3**. The mixture underwent sep-pak purification to afford the pure **ODN3**.

MALDI-TOF MS (*m*/*z*) **ODN2 (SOMe)**: [M-H]^−^ calcd. 4060.9; found 4060.6.

#### ODN3(vinyl)

[M-H]^−^ calcd. 3898.6; found 3898.2.

### General procedure for the crosslink reactions

The reaction was performed with 10 μM **ODN3** and 5 μM target DNA or RNA labeled with fluorescein at the 5′-end in a buffer of 100 or 500 mM NaCl and 50 mM MES at pH 7.0. The reaction mixture was then incubated at 37°C. An aliquot of the reaction mixture was collected at each time and quenched by adding loading dye (95% formamide, 20 mM EDTA, 0.05% xylene cyanol and 0.05% bromophenol blue). The crosslinked products were analyzed by denaturing 20% polyacrylamide gel electrophoresis containing urea (7 M) with TBE buffer at 300 V for 1 h. The labeled bands were visualized and quantified with a fluorescent image analyzer (FLA-5100FujiFilm). The crosslink yield was calculated from the ratio of the crosslinked product to the remaining single-stranded DNA or RNA.

### Isolation of the crosslinking adducts and preparing the NMR sample

The crosslinking reaction was performed with the reactive **ODN3** (200 μM) and the complementary DNA**1** (**N** = dG or 8-oxoG) (100 μM) in a buffer (100mM NaCl, 50 mM MES buffer, pH 7.0) at 37°C. After 72 h for DNA**1** (**N** = dG) or 24 h for DNA**1** (**N** = 8-oxoG), each crosslinked adduct was purified by HPLC using an ODS column (Nacalai Tesque: COSMOSIL 5C18-MS-II, 10 × 250 mm) by a linear gradient of 10–40%/20 min of acetonitrile in 0.1 M TEAA buffer at a flow rate of 4 ml/min. After isolation of each crosslinked adduct by RP-HPLC, the remaining triethyl ammonium was completely removed by ethanol precipitation and gel filtration (NAP-25 column, GE-Healthcare) MALDI-TOF MS (*m*/*z*) guanine adduct (**10**): calcd. 7924.2, found 7924.6. 8-oxoguanine adduct **(11)**: calcd. 7940.2, found 7944.0.

## RESULTS

### AOVP ODN synthesis and crosslinking reactivity evaluation

**Scheme 1.**
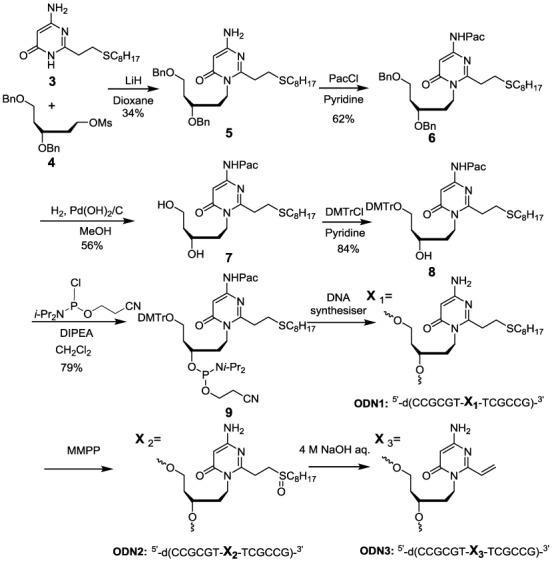
Synthesis of the ODN**3**.

The synthesis of ODN containing **2** is summarized in Scheme 1. Base (**3**) was synthesized as previously described (42). The acyclic side chain (**4**) was synthesized from 2′-deoxy-D-ribose by modifying a reported procedure (43,44). The coupling reaction of **3** and **4** provided the desired N1 alkylated product (**5**) using LiH as a base in 1, 4-dioxane. The low yield of this reaction may be attributed to the low reactivity of **3** (42). The structure of **5** was confirmed by 1D-NMR, 2D-NMR and high-resolution mass spectrometry. The amino group of **5** was protected by a phenoxyacetyl group to give **6**. Diol (**7**) was obtained by removing the benzyl groups by hydrogenation with Pd(OH)_2_/C. After protection of the primary hydroxyl group with dimethoxytrityl, **8** was converted to the phosphoramidite building block (**9**) for the ODN synthesis. Sulfide-protected ODN1 was obtained by using **9** in an automated DNA synthesizer and purifying by RP-HPLC. After oxidizing ODN**1** to sulfoxide ODN**2** with magnesium monoperphthalate (MMPP), elimination of the sulfoxide under alkaline conditions afforded ODN**3** (Scheme 1). The molecular weight of ODN**3** was confirmed by MALDI-TOF mass spectroscopy. ODN**3** was purified with a Sep-Pak cartridge and could be stored for months at -20°C with no decomposition of the sequence.

**Figure 2.**
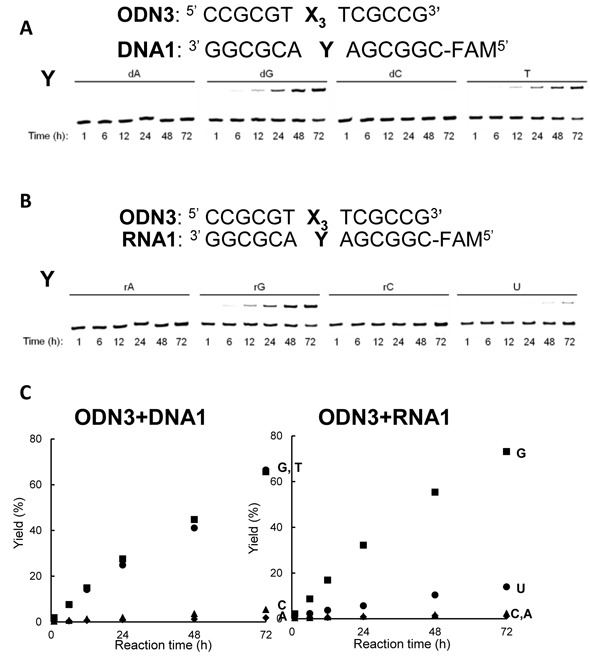
Crosslinking reaction of **ODN3** with **DNA1** and **RNA1** (**A**) The denaturing gel electrophoresis for the crosslinking reaction to DNA. (**B**) The denaturing gel electrophoresis for the crosslinking reaction to RNA. (**C**) A summary for the time course of the crosslink yield, **Y** = A; ♦, **Y** = G; ▪, **Y** = C; ▴, **Y** = T or U; •. The reaction was performed using 10 μM ODN**3** and 5 μM DNA**1** or RNA**1** in 100 mM NaCl, 50 mM MES, pH 7 at 37ºC.

The crosslinking reactivity of ODN**3** toward the complementary DNA**1** and RNA**1** labeled with fluorescein at the 5′ end was evaluated under neutral conditions (Figure 2).

The reactions were analysed by 20% polyacrylamide denaturing gel electrophoresis. Figure 2A and B illustrate the reactivity of ODN**3** toward the different bases at the target site in DNA**1** and RNA**1**. The slower mobility bands corresponding to the crosslinked product were observed in the reaction between ODN**3** and DNA**1** (**Y** = G or T) or RNA**1** (**Y** = G or U).

**Figure 3.**
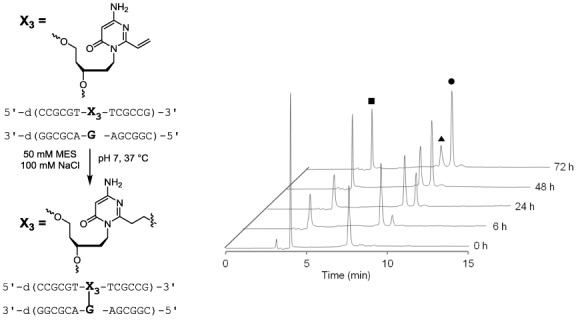
HPLC trace for the crosslinking reaction between **ODN3** and **DNA1** (Y = G). The reaction was performed with 10 μM **ODN3** and 5 μM **DNA1** in 100 mM NaCl, 50 mM MES, pH 7 at 37ºC. ▪: ODN (Vinyl), ▴: FAM labelled target DNA, •: crosslink adduct. HPLC conditions: COSMOSIL 5C18-MS-II (4.6 × 250 mm), solvent A: 0.1 M TEAA buffer (pH 7), B: CH_3_CN, B: 10%–40%/20 min, 40%–100%/30 min, linear gradient, flow rate: 1.0 ml/min, UV-monitor: 254 nm, 50ºC.

The crosslink yields were calculated from the ratio of the slower mobility band of the crosslinked product to the sum of the crosslinked product and the remaining single stranded bands. The time course of the crosslink yields for DNA**1** and RNA**1** is summarized in Figure 2C. There were no significant differences in the crosslink yields when the crosslinking reactions were carried out in 1:1 ratios of ODN**3** versus target DNA or RNA (Supplementary Figure S2). A similar crosslinking reactivity with ODN**3** was observed for guanine and thymine in DNA**1**. However, the crosslink yield for guanine in RNA**1** was higher than that for uracil. Thus, ODN**3** showed a different base selectivity in DNA and RNA. No significant difference in the crosslink yield was observed for uracil (U) and 5-methyl uracil (rT) in RNA (Supplementary Figure S4). These results were consistent with the change in the base selectivity in DNA and RNA arising from the difference in the duplex conformation between DNA-DNA and DNA-RNA, rather than to the difference between uracil and thymine. Theoretical analysis of the DNA-DNA and DNA-RNA duplexes has shown that the flexibility of the DNA-RNA hybrids is lower than that of the DNA-DNA duplex (45). AOVP with an acyclic linker might be in close proximity to both thymine and guanine in the duplex DNA, which is a flexible complex. However, AOVP may not have access to uracil in the DNA-RNA hetero duplex with a less flexible structure and selectively reacted with guanine. To gain more insight into the crosslinking reaction between ODN**3** and DNA**1** (**Y** = dG), the reaction was also analyzed by HPLC (Figure 3). After 72 h, a new peak corresponding to the crosslinked adduct was observed in addition to the ODN**3** and DNA**1** (**Y** = dG).

The intensity of the new peak increased with reaction time and the intensity of the ODN**3** and DNA**1** peaks decreased. The new peak was isolated by HPLC, and the isolated compound was found to have a mass consistent with a crosslinked duplex DNA (calcd.[M-H] 7940.2 found [M-H] 7944.0).

It was confirmed that the crosslinking reactions occurred at the opposing guanine in the target strand by hydroxyl radical cleavage of the purified crosslinked product (46). The NMR analyses of the product are described below.

#### Effect of metal ions on crosslinking reactivity

**Figure 4.**
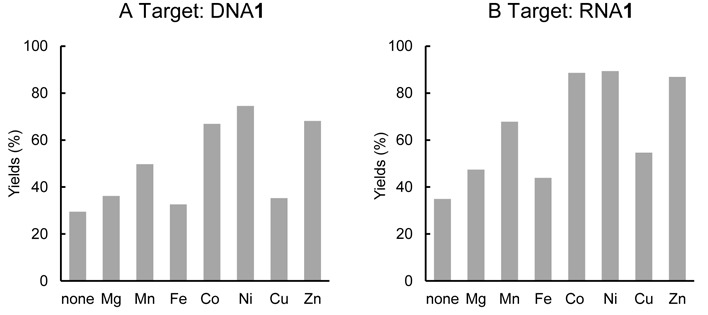
Crosslinking reactions of ODN**3** with DNA**1** (**A**) and RNA**1 (B)** (**Y** = G) in the presence of metal ions. The reactions were performed under the same conditions as described in Figure 2 with 1 mM metal ions except for CuCl_2_ (0.1 mM). Each mixture was incubated for 24 h and analyzed by denaturing gel electrophoresis.

In the crosslinking reactions between ODN**3** and DNA**1**, we observed that the selectivity to guanine was slightly increased under alkaline conditions. The nucleophilicity of the guanine base is closely related to the pH, and the nucleophilicity of N1 and O6 **i**ncreases at pH > 9 (47,48). Furthermore, the addition of transition metals, which coordinate at the N7 position of the guanine base, increases the nucleophilic reactivity of guanine at N2 (49). Next, we investigated the effect of the metal dichloride on the crosslinking reactions between ODN**3** and DNA**1** (**Y** = dG), RNA**1** (**Y** = G). The crosslink yields in the presence of a 1 mM metal cation, except for CuCl_2_ (0.1 mM), after 24 h are summarized in Figure 4. A large increase in the reaction yields for guanine in DNA**1** and RNA**1** was observed in the presence of CoCl_2_, NiCl_2_, ZnCl_2_ or MnCl_2_. The metal ion effect was greater in RNA than in DNA.

**Figure 5.**
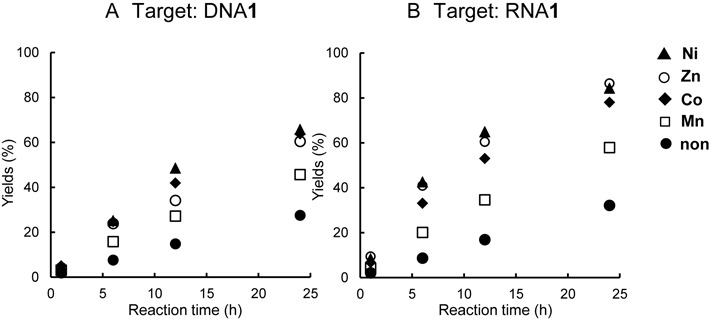
Effect of metal cations on the crosslinking reaction rate. The reactions were performed under the same conditions as described in Figure 2 with 1 mM NiCl_2_, ZnCl_2_, CoCl_2_ and MnCl_2_. After the incubation for the indicated time, each mixture was analyzed by denaturing gel electrophoresis.

The crosslinking reaction rate to DNA**1** and RNA**2** was also investigated in the presence of CoCl_2_, NiCl_2_, ZnCl_2_ or MnCl_2_. The rates of the crosslinking reaction with guanine were increased by adding metal cations (Figure 5). The remarkable acceleration in the reaction with the RNA target was observed in the presence of Ni, Zn and Co and the yields reached 80% within 24 h. The crosslinking reactions with DNA were also accelerated by adding Ni^2+^, Zn^2+^,Co^2+^ and Mn^2+^.

**Figure 6.**
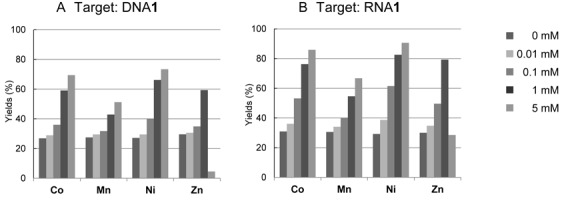
Concentration dependencies of the metal cations on the crosslinking reactions. The reactions were performed under the same conditions as described in Figure 2 with NiCl_2_, ZnCl_2_, CoCl_2_ and MnCl_2_. After the incubation for 24 h, each mixture was analysed by denaturing gel electrophoresis.

The concentration dependence of CoCl_2_, MnCl_2_, NiCl_2_ and ZnCl_2_ on the crosslink yields with ODN**3** and DNA**1** or RNA**1** was investigated (Figure 6). The crosslink yields increased with the increasing concentration of CoCl_2_, MnCl_2_ and NiCl_2_. However, for ZnCl_2_, the crosslink yield reached a maximum at 1 mM. Short ODNs can be precipitated with Zn^2+^ ions at a concentration higher than 5 mM (50) and the low reactivity in the presence of 5 mM Zn^2+^ ions was attributed to the formation of a precipitate.

**Figure 7.**
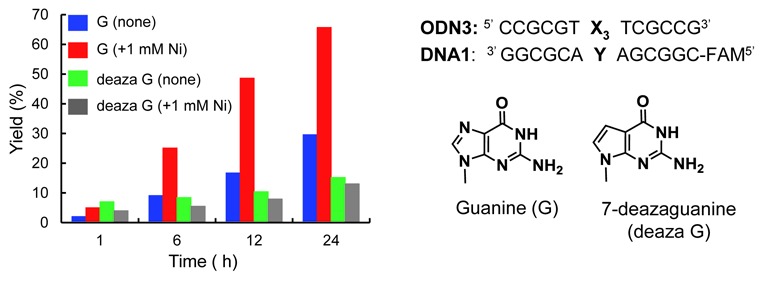
Comparison of the crosslinking reactivity to dG and deaza dG in the presence or absence of 1 mM Ni^2+^. The reactions were performed under the same conditions as described in Figure 2 with 1 mM NiCl_2_.

Next, we investigated the effect of the nucleoside structure on the activation by the addition of Ni. The coordination at the N7 position of guanine was expected to play an important role in the activation. To gain an insight into this reaction, we compared the reactivity of 7-deazaguanine (deaza dG), which lacks the N7 nitrogen atom, with that of guanine, both in the presence and absence of Ni^2+^ (Figure 7). The crosslinking reaction with dG was accelerated by the addition of Ni^2+^. However, the reactivity of deaza dG was lower than that of dG, and was not affected by the addition of Ni^2+^.

#### Crosslinking reactivity with purine derivatives

The addition of metal cations accelerated the crosslinking reactions with ODN**3**. Based on these results, we hypothesized that the pKa value of the N1 position in guanine might affect the crosslink reactivity, because the transition metals involved in accelerating the crosslinking reaction coordinate at the N7 position of the guanine base and decrease the pKa of the N1 position (51). Thus, we explored the crosslinking reactions of ODN**3** with the guanine analogs, inosine (I), 8-oxoguanine (8-oxoG) and 2-aminopurine (2-AP). Inosine and 8-oxoG have different pKa(N1) values.

The crosslinking reactions between ODN**3** and DNA**1** (**Y** = G, 8-oxoG, I, 2-AP) were performed under the same conditions as described in Figure 2. Each reaction was analyzed with 20% polyacrylamide denaturing gel electrophoresis and the crosslink yields were calculated based on these results.

**Figure 8.**
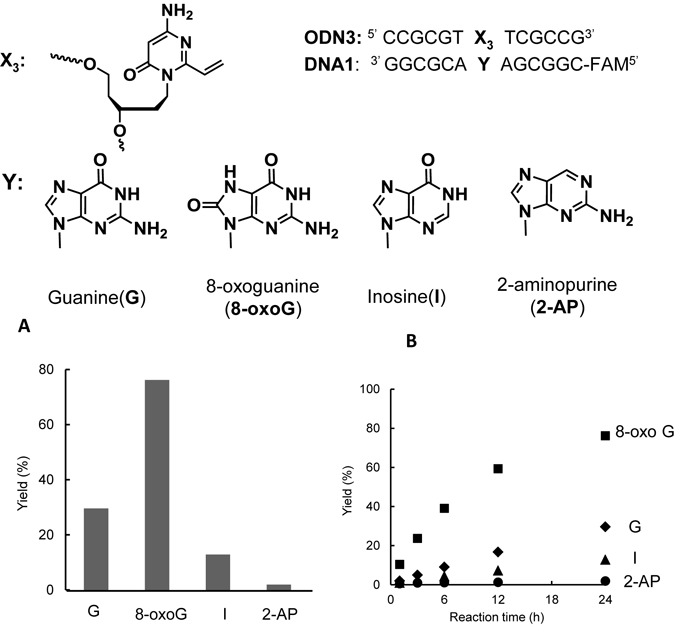
Crosslinking reactivity of ODN**3** with DNA**1** containing guanine derivatives. The reactions were performed under the same conditions as described in Figure 2. Each mixture was incubated for 24 h (**A**) or 0–24 h (**B**) and analyzed by denaturing gel electrophoresis.

Figure 8 shows the crosslinking reactivity of ODN**3** with DNA containing guanine derivatives. After 24 h, the rank order of the crosslink yields was 8-oxoG > guanine > inosine > 2-AP. The crosslink rate of ODN**3** to 8-oxoG is faster than that of guanine (Figure 8B). The pKa (N1) of guanine and 8-oxoG is reported to be 9.6 and 8.9, respectively (52). As we expected from the pKa (N1) value, the highest crosslinking reaction efficiency was observed with 8-oxoG. To examine the effect of the duplex stability on the efficiency of the crosslinking reactions, the melting temperature (T*_m_*) values were measured for each duplex. For this purpose, the vinyl group of ODN**3** was reduced with NaBH_4_ to give a non-reactive ODN that had an ethyl group instead of a vinyl group. The T*_m_* values for the duplexes formed between the reduced ODN**3** and target DNA containing guanine derivatives (**Y** = G, 8-oxoG, I, 2-AP) were determined to be 43.8°C (**Y** = G), 48.5°C (**Y** = 8-oxoG), 49.8°C (**Y** = I) and 54.0°C (**Y** = 2-AP) (Supplementary Table S1). Although the lowest reactivity was observed for 2-AP, the thermal stability of the duplex between ODN**3** and DNA**1** (**Y** = 2-AP) was the highest. The differences in the thermal stability could not explain the efficiency of the crosslinking reactions. We discuss on the differences between the reactivity with inosine and 2-AP in the Discussion section.

#### Structural determination of the crosslink products

To elucidate the structure of the reaction site on the guanine and the 8-oxoG bases, we next purified each crosslink adduct (**10** and **11**) in the reactions between ODN**3** and DNA**1** (**Y** = dG or 8-oxoG) by RP-HPLC. We tried enzymatic hydrolysis of the purified crosslink adduct between ODN**3** and DNA**1** (**Y** = 8-oxoG) to determine the structure; however, we could not isolate the hydrolyzed product because the decomposition of the crosslink adduct through the further oxidation of 8-oxoG occurred during the enzymatic hydrolysis reaction. Therefore, we used high-resolution NMR spectra to determine the structure of the crosslinked duplex DNA. After purifying the crosslinked adduct, the remaining triethyl amine was completely removed by ethanol precipitation and subsequent gel filtration (NAP-25 column).

**Figure 9.**
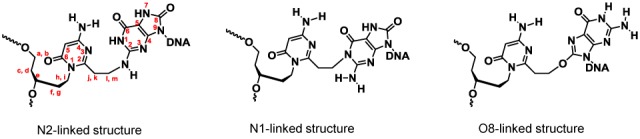
Proposed structures for the adduct to 8-oxoguanine.

The potential crosslinking sites are N1 and N2 of G/8-oxoG, and O8 of 8-oxoG, resulting in the N1-, N2- and O8-linked products, respectively (Figure 9). High-resolution NMR spectroscopic investigations were performed to determine the structure of each crosslink adduct. Sequential NOE assignments of the crosslink oligonucleotides were made from the H6/H8-H1’ fingerprint regions in the 2D NOESY spectra in H_2_O and/or D_2_O using standard methods (53–55). Cytosine H5 and adenine H2 were assigned by the strong H5-H6 NOEs and long range H2-H1’ NOEs, respectively. Based on the unambiguous cytosine H5 and adenine H2 signals, the labile guanine imino H1 and thymine imino H3 involved in forming the Watson-Crick base pairs were assigned using the cytosine H5-H41/H42 and cytosine H41/H42-guanine H1 NOEs, and adenine H2-thymine H3 NOEs (56). Other sugar protons including H2’, H2’’, H3’ and linker protons were assigned based on a combination of 2D TOCSY and DQF-COSY with the supporting information from the NOESY and ^1^H-^13^C HSQC spectra.

**Figure 10.**
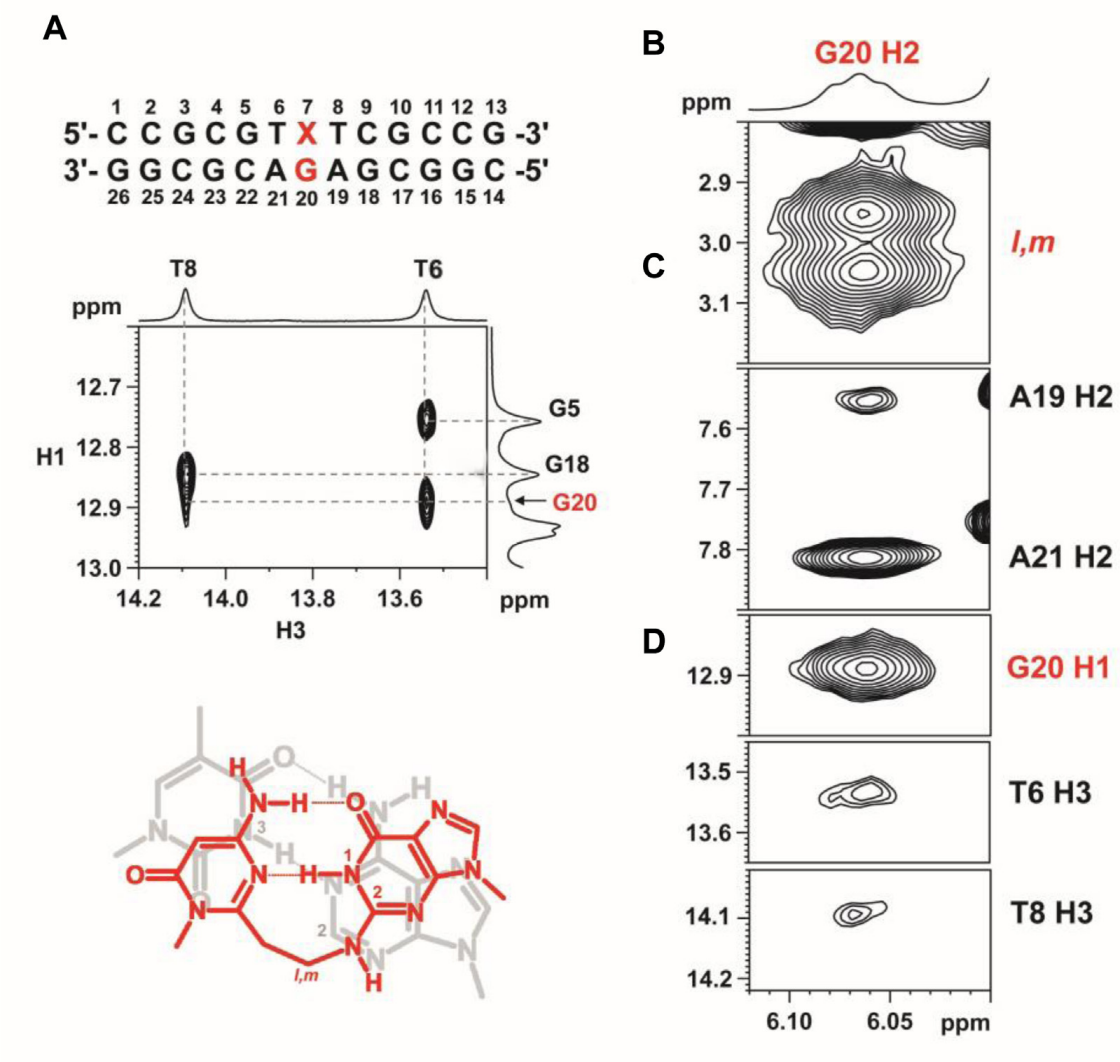
Structural analysis of a crosslink product between ODN**3** and DNA**1** (**Y** = G). **(A)** The presence of G20 H1-T6/T8 H3 NOEs suggest the formation of X7-G20 base pair (red), which stacks well between the flanking T6-A21 and T8-A19 base pairs. For clarity, only T6-A21 (gray) was shown. G20 H2 shows NOEs with **(B)** linker protons*l* and *m*, **(C)** A19/A21 H2, **(D)** G20 H1 and T6/T8 H3. NOESY was acquired at 15°C and a mixing time of 300 ms.

For the crosslink product (**10**) between ODN**3** and DNA**1** (**Y** = dG), one set of signals was observed and complete sequential assignments were found for residues C1 to T6 and T8 to G13 of ODN**3**, and C14 to G26 of DNA**1** (Supplementary Figure S6 in SI). The presence of NOEs between G20 H1 and T6/T8 H3 in the NOESY spectrum at 15°C suggests G20 forms a base pair with the AOVP residue (X7) and the base pair remains stacked between the flanking T6-A21 and T8-A19 Watson-Crick base pairs (Figure 10A). By comparing the NOESY spectra in H_2_O and D_2_O, the G20 H2 labile proton was found at 6.06 ppm at 15°C. This signal shows strong NOEs with the linker protons*l* and *m*, and its assignment was further supported by the NOEs with (i) G20 H1, and (ii) A19/A21 H2 and T6/T8 H3 of the flanking TA base pairs (Figure 10, B–D). The presence of both G20 H1 and H2 indicates that the crosslinking reaction occurred at the N2 position of G20 between ODN**3** and DNA**1** (**Y** = dG).

**Figure 11.**
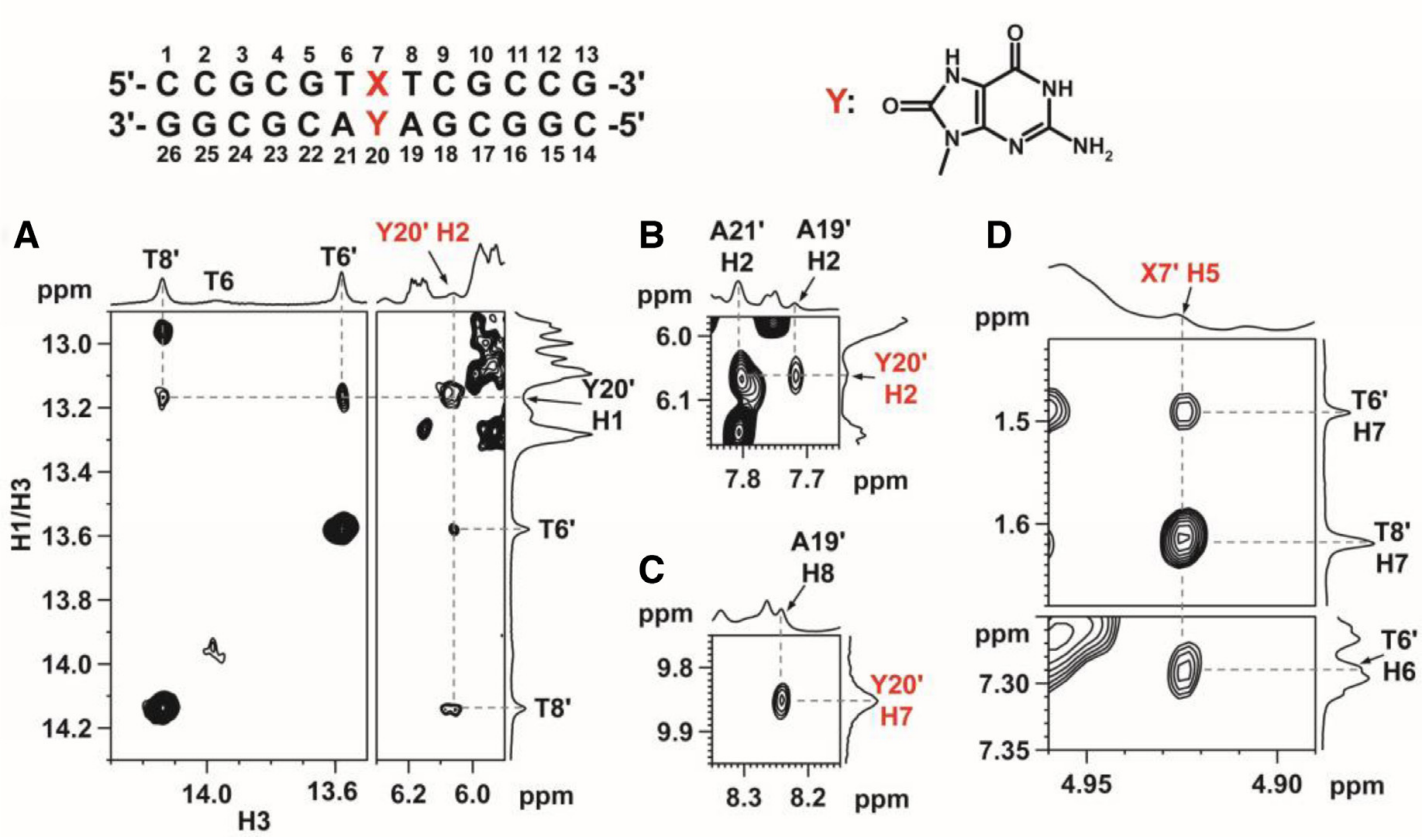
Characteristic NOEs of the prime signals of a crosslink product between ODN**3** and DNA**1** (Y = 8-oxoG). **(A**) Y20’ H1-T6’ H3, Y20’ H1-T8’ H3, Y20’ H1-Y20’ H2, Y20’ H2-/T6’ H3 and Y20’ H2-T8’ H3. **(B)** Y20’ H2-A19’ H2 and Y20’ H2-A21’ H2. **(C)** Y20’ H7-A19’ H8. **(D)** X7’ H5-T6’ H7, X7’ H5-T8’ H7 and X7’ H5-T6’ H6. For (A)–(C), NOESY was acquired in 90% H_2_O/10% D_2_O at 5°C and a mixing time of 280 ms. For (D), NOESY was acquired in100% D_2_O at 40°C and a mixing time of 350 ms.

In the crosslink product (**10**), T6-A21 and T8-A19 are the only two TA base pairs, therefore two sets of thymine and adenine signals were observed. Surprisingly, when Y20 is 8-oxoG, the ^1^H-^13^C HSQC spectra showed four adenine and thymine signals. These signals did not merge into two upon increasing the temperature to 90°C, suggesting the presence of two crosslink products instead of one with two different conformations. As a result, two sets of NMR signals were obtained for the crosslink products (**11**) between ODN**3** and DNA**1** (**Y** = 8-oxoG). To differentiate the two sets of signals, a prime symbol was added as a suffix to the residue number for the second set of signals. Sequential assignments were also made from the 2D NOESY fingerprint regions in H_2_O and/or D_2_O. In general, sequential NOEs were found for residues from C1 to T6 and G10 to G13 of ODN**3** and C14 to C17 and A21 to G26 of DNA**1** in both sets of signals. Their chemical shifts follow quite well with the prediction results for B-DNA double helices (57,58). However, large chemical shift differences were observed between T8 and T8’. The methyl H7 and aromatic H6 chemical shifts of T8’ were similar to those observed in B-DNA, but they were both downfield shifted in T8, appearing in regions similar to those observed in the unstructured DNA (59). For the ‘prime’ signals, the NMR features are similar to those observed in the N2-linked product between ODN**3** and DNA**1** (**Y** = G). The NOEs between Y20’ H1 and T6’/T8’ H3 in the NOESY spectrum at 5°C (Figure 11A) suggest Y20’ forms a base pair with X7’ and the base pair remains stacked between the flanking T6’-A21’ and T8’-A19’ Watson-Crick base pairs.

By comparing the NOESY spectra in H_2_O and D_2_O, the Y20’ H2 labile proton was found at 6.05 ppm at 5°C. This signal shows strong NOEs with the linker protons *l* and *m*. The assignment of Y20’ H2 was further supported by the NOEs with (i) Y20’ H1, and (ii) T6’/T8’ H3 (Figure 11A) and A19’/A21’ H2 of the flanking TA base pairs (Figure 11B). These, together with the Y20’ H7-A19’ H8 NOEs, further support the base stacking (Figure 11C). Upon raising the temperature to 40°C, X7’-Y20’ remains stacked as X7’ H5 still shows NOEs with T6’/T8’ H7 and T6’ H6 (Figure 11D). The presence of both Y20’ H1 and H2 reveals that the ‘prime’ signals belong to the N2-linked structure.

**Figure 12.**
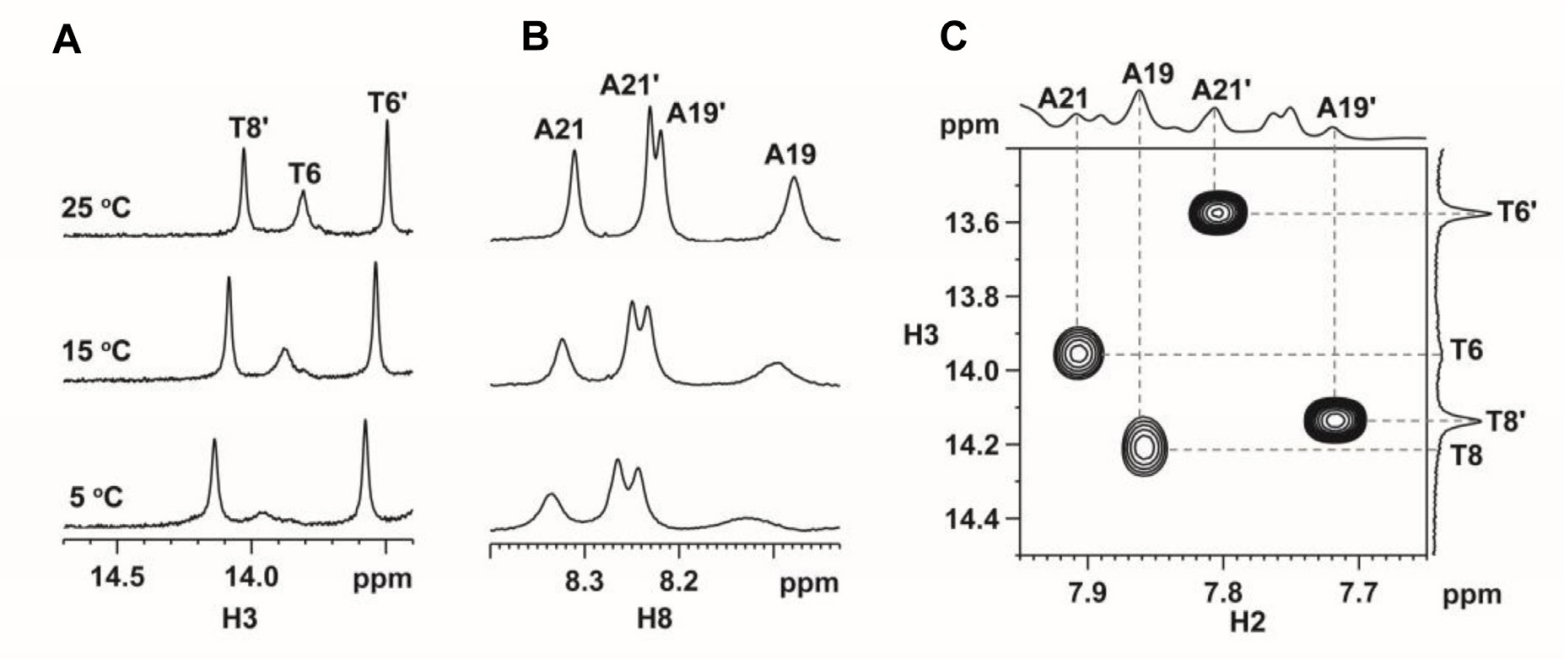
Variable temperature ^1^H NMR spectra of **(A)** H3 imino, and **(B)** H8 aromatic protons. **(C)** T8 H3-A19 H2 NOE was observed at 5°C.

Based on the sequential assignment results for the set of ‘no prime’ signals, the T8 H6 and H7 chemical shifts were found to be unusually downfield when compared to those in B-DNA, but similar to those in the unstructured DNA. The T6 H3 imino signal could be observed but became significantly broadened (Figure 12A) when the temperature was lowered to 5°C. Such broadening was also found in A19 H8 (Figure 12B), suggesting the presence of a conformational exchange involving the middle three base pairs. At 5°C, an NOE was observed between T8 H3 and A19 H2, but not at 10°C or above (Figure 12C), suggesting T8-A19 base pair was present in one conformer at 5°C.

In order to determine whether the ‘no prime’ signals belonging to the N1-linked structure, we attempted to look for NOEs between the Y20 H2 amino protons and the linker protons (*l* and *m*). Unfortunately, no NOE was observed, therefore, no evidence could be obtained to differentiate the signals belonging to the N1-linked or O8-linked structures. The absence of Y20 H2 amino-linker NOEs in the N1-linked structure may result from line broadening due to a moderate conformational exchange that occurs at lower temperatures. Although increasing the temperature can help to reduce line broadening, the exchange rate of the amino protons with water will increase, making the detection of NOEs with these amino protons difficult.

**Figure 13.**
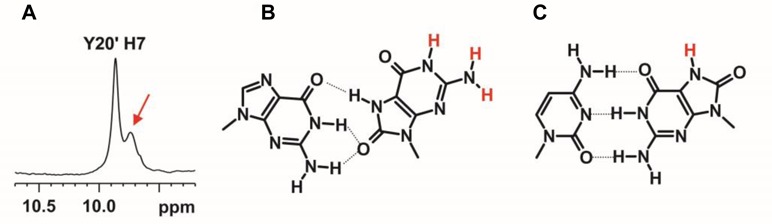
**(A)** An unassigned imino signal was observe at 9.72 ppm at 5°C (indicated by the red arrow). **(B)** A stable G(*anti*)-8-oxoG(*syn*) base pair, similar to the O8-linked structure, was found well-stacked between the Watson-Crick flanking base pairs (60). Both the 8-oxoG H1 and H2 (in red) were not detected as they were exposed to solvent and not hydrogen-bonded. **(C)** A Watson-Crick C-8-oxoG base pair was found to form in a double-helix (61). The 8-oxoG H7 (in red) was also exposed to solvent and not hydrogen-bonded but it could be observed between 9.5 and 10 ppm at temperatures below 40°C.

Even though no direct spectral evidence was obtained to determine the type of crosslinks, there was an unassigned guanine imino signal at 9.72 ppm at 5°C (Figure 13A) in which its identity of being an H7 or H1 imino proton serves as an important clue to determine whether this is N1-linked or O8-linked. In the NMR structural investigation on the effect of 8-oxoG opposite to a G in a double helix, a stable G(*anti*)-8-oxoG(*syn*) base pair was found well-stacked between the Watson-Crick flanking base pairs (60). The 8-keto group was determined to interact with the opposite G imino and amino protons, making the environment of the amino and imino groups of 8-oxoG similar to that of the O8-linked structure (Figure 13B). Although the G(*anti*)-8-oxoG (*syn*) base pair was well-stacked, both the 8-oxoG H1 imino and H2 amino protons were not detected as they were exposed to solvent and not hydrogen-bonded. As a result, it is unlikely that the observed unassigned guanine imino signal belongs to an H1 signal. Meanwhile, another solution NMR structural investigation revealed that 8-oxoG and C formed a Watson-Crick base pairing in a double-helix (61). Although the H7 imino proton was also exposed to solvent and not hydrogen-bonded (Figure 13C), this proton could be observed between 9.5 and 10 ppm at temperatures below 40°C. As a result, the signal at 9.72 ppm was assigned to the Y20 H7 imino proton, suggesting the presence of the N1-linked structure in the crosslink products (**11**).

#### Ratio of the two crosslink products to 8-oxoG

The 1D ^1^H NMR spectrum with a 78 s recycling delay was performed to assure full magnetization recovery for accurate peak integral measurements (Supplementary Figure S7). As the aromatic H8 signals of A19 and A21 were well-resolved from those of A19’ and A21’, their peak integrals were measured and the ratio of the N2-linked to N1-linked species were found to be 1:1 for the crosslink product (**11**) of ODN**3** and DNA**1**(**Y** = 8-oxo-dG).

## DISCUSSION AND CONCLUSION

**Figure 14.**
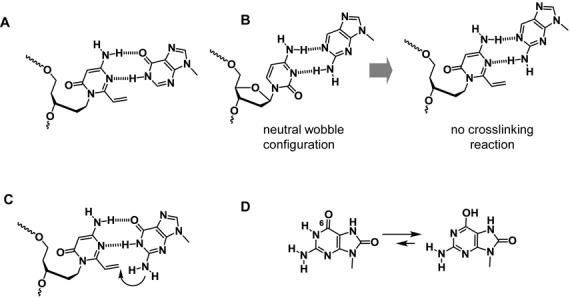
**(A–C)** Speculative complex structure between AOVP and guanine derivatives. (**D**) Tautomerization of 8-oxoG.

We have synthesized ODN containing AOVP with an acyclic linker. These ODNs exhibited crosslinking reactivity with guanine and thymine in DNA and guanine in RNA. In the reactions between AOVP and guanine derivatives, a highly efficient reaction with 8-oxoG and lower reactivity with inosine and 2-aminopurine were observed in comparison with guanine. High-resolution NMR analysis of the adduct between AOVP and guanine demonstrated that the crosslinking reaction occurred at the N2 position of guanine. However, AOVP reacted with 8-oxoG at the N1 and N2 positions in an equal ratio. Based on the structural analysis, we can rationalize the crosslinking reaction results between AOVP and the guanine derivatives. The lower reactivity with inosine could be attributed to the absence of the 2-amino group and fixed by hydrogen bonding formation in the non-reactive structure (Figure 14A). The 2-AP-cytosine base pair is dominant in a neutral wobble configuration at physiological pH (Figure 14B) (62). The AOVP and 2-AP base pair may form a similar configuration with the 2-AP-cytosine base pair, and this configuration could cause the low reactivity of 2-AP with AOVP.

However, guanine can form a complex with AOVP as shown in Figure 14C, resulting in high selectivity because of the close proximity of the 2-amino group of guanine to the vinyl group of AOVP. The pKa value of the N1 position in 8-oxoG is reported to be 8.6 and the 6-enol form exists at physiological pH (63). The investigation of the relative stabilities of the tautomers of 8-oxoG and guanine by calculation suggested that the population of the 6-enol form in 8-oxoG is higher than that in guanine (64).

**Figure 15.**
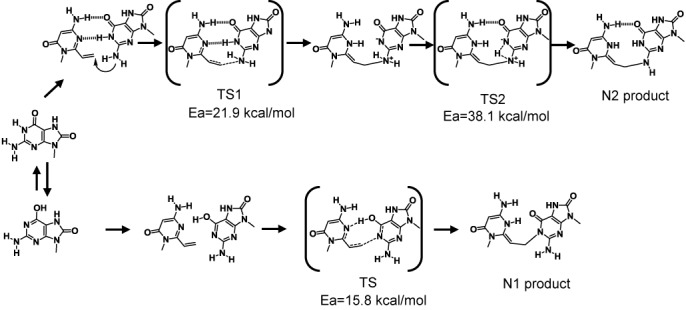
Calculated pathway of crosslinking with AOVP to 8-oxoG and estimated activation energy of the transition states by *ab initio*.

Based on these reports, we hypothesize that the reaction between AOVP and 8-oxoG proceeds via two pathways to produce the N1 and N2-adducts, and we performed *ab initio* molecular orbital calculations to elucidate the mechanism. The reaction for producing the N2 adduct may proceed via two transition states (TS1, TS2) and the activation energy is 21.9 kcal/mol and 38.1 kcal/mol, respectively. However, the alternative mechanism with the 6-enol tautomer of 8-oxoG for producing the N1-adduct provided a transition state with a lower activation energy (15.8 kcal/mol). Because the abundance of the 6-enol form is higher for 8-oxoG than for guanine, the pathway may account for the efficient crosslinking reactions between AOVP and 8-oxoG. However, these calculations do not explain the ratio of the adducts for 8-oxoG and further studies are necessary (Figure 15).

In conclusion, we have developed AOVP with an acyclic spacer as a crosslinking nucleobase that shows the crosslinking reactivity with guanine. In addition, the AOVP CFO reacted with 8-oxoG opposite AOVP with a higher efficiency than with guanine. To our knowledge, this is the first example of the selective crosslinking reaction with 8-oxoG.

We show for the first time the direct structural determination of adducts in the duplex DNA without enzyme digestion. The covalently linked duplex DNA structure with our crosslinking reaction may provide new tools for investigating DNA repair mechanisms.

## Supplementary Material

SUPPLEMENTARY DATA
