# Supplementary material for: Crosslinking reactions of 4-amino-6-oxo-2-vinylpyrimidine with guanine derivatives and structural analysis of the adducts
Source: Nucleic Acids Res. 2015 Aug 5;43(16):7717–30. doi: 10.1093/nar/gkv797 (PMC4652779; doi:10.1093/nar/gkv797)
Supplement: SUPPLEMENTARY DATA [file supp_43_16_7717__index.html]

Crosslinking reactions of 4-amino-6-oxo-2-vinylpyrimidine with guanine derivatives and structural analysis of the adducts — Crosslinking reactions of 4-amino-6-oxo-2-vinylpyrimidine with guanine derivatives and structural analysis of the adducts — SUPPLEMENTARY DATA 

# Crosslinking reactions of 4-amino-6-oxo-2-vinylpyrimidine with guanine derivatives and structural analysis of the adducts

## SUPPLEMENTARY DATA

- SUPPLEMENTARY DATA
