# Supplementary material for: Crosslinking reactions of 4-amino-6-oxo-2-vinylpyrimidine with guanine derivatives and structural analysis of the adducts
Source: Nucleic Acids Res. 2015 Aug 5;43(16):7717–30. doi: 10.1093/nar/gkv797 (PMC4652779; doi:10.1093/nar/gkv797)
Supplement: SUPPLEMENTARY DATA [file supp_gkv797_nar-01472-f-2015-File004.pdf]

Supporting Information: Crosslinking Reactions of 4-Amino-6-oxo-2-vinyl pyrimidine (AOVP) with Guanine Derivatives and Structural Analysis of the Adducts

Shuhei Kusano[1], Shogo Ishiyama[1], Sik Lok Lam[2*], Tsukasa Mashima[3], Masato Katahira[3], Kengo Miyamoto[4], Misako Aida[4], Fumi Nagatsugi[1*]

[1]Institute of Multidisciplinary Research for Advanced Materials, Tohoku University, 2-1-1 Katahira, Aoba-ku, Sendai-shi, 980-8577, Japan.

[2]Department of Chemistry, The Chinese University of Hong Kong, Shatin, New Territories, Hong Kong

[3]Institute of Advanced Energy, Graduate school of Energy Science, Kyoto University

[4]Department of Chemistry, Graduate School of Science, Hiroshima University

Experimental

·General

All air sensitive reactions were carried out under argon in oven-dried glassware using standard syringe and septa techniques, unless otherwise noted. The ^{1}H and ^{13}C NMR were recorded on a JEOL LAMBDA 400 (400 MHz for 1H and 100 MHz for ^{13}C), a JEOL LAMBDA 600 (600 MHz for 1H and 150 MHz for 13C), Bruker 400 (400 MHz for ^{1}H and 100 MHz for ^{13}C) or a Bruker 500 (500 MHz for 1H and 125 MHz for ^{13}C) spectorometer using Chloroform (^{1}H, δ = 7.26), MeOH (^{1}H, δ = 3.34), DMSO (^{1}H, δ= 2.54) and $CDCl_3$ (^{13}C, δ = 77.0) as an internal standards. ^{31}P NMR were recorded on a JEOL LAMBDA 400 (162 MHz for ^{31}P) or Bruker 400 (162 MHz for ^{31}P) using H_3PO_4 (85%) as an external standard. Multiplicity and qualifier abbreviations are as follows: s = singlet, d = doublet, t = triplet, q = quartet, quint. = quintet, sept. = septet, m = multiplet, br = broad. ESIMSs were recorded using a BioTOF II mass spectrometer. High resolution mass analyses (HRMS) were recorded on APEX Ⅲ (Bruker Daltonics) or MicrOTOFQII mass spectrometer. MALDI-TOF mass spectra were measured using Autoflex speed mass spectrometer and the laser at 337 nm by negative mode using 3-hydroxypicolinic acid as the matrix or positive mode using 2,5-dihydroxybenzoic acid as the matrix. Thin-layer chromatography was performed on Merck 60 F254 precoated silica gel plates. Merck 60 F254 precoated silica gel on glass in a thickness of 0.9 mm was used for preparative TLC. Column chromatography was performed on silica gel (Silica Gel 60 N; 63–210 mesh, KANTO CHEMICAL CO., INC. or 40–50 mesh, KANTO CHEMICAL CO., INC.). The ultraviolet-visible (UV-vis) absorption spectra were recorded by a BECKMAN COULTER DU800. ODN synthesis was carried out by the use of an automated DNA synthesizer (ABI, 392 DNA/RNA synthesizer) following

the standard phosphoramidite chemistry. High performance liquid chromatography (HPLC) was performed using nacalai tesque cosmosil 5C18MS (4.6 or 10 × 250 mm) as the columns, JASCO PU-986 as the pump, JASCO 2075 as the UV monitoring, and JASCO 2067 as the column oven. pH measurements were measured performed on Mettler Toledo Seven Easy pH meter using ORION 8220BNWP as the electrode. PCR was carried out using Biorad Mycycler Thermal Cycler. Luciferase activity was analyzed by GloMax 20/20 luminometer (promega). Densitometric analysis of the gel was carried out on the 20% denaturing polyacrylamide gel plates, and visualized, quantified with use of a FLA-5100 Fluor Imager. Anhydrous DMF, MeOH, THF, CH_2Cl_2, dioxane, pyridine, toluene and THF were purchased from Wako Pure Chemical Industries Ltd. and stored over molecular sieves under argon. Et_3N and DIPEA were dried and stored over KOH under argon atmosphere. Commercial available reagents were obtained from Wako Pure Chemical Industries Ltd., KANTO CHEMICAL CO., INC. and used without further purification. DNA and RNA oligomer was purchased from Japan Bio Services Co., LTD. (Saitama, JAPAN), buffers and salts was purchased from Nakalai Tesque.

4-amino-2-(2-octylthioethyl)-6-oxo-1-(3’,5’-*O*-dibenzyl-2’,4’-dideoxy-D-ribityl)pyrimidine (5)

^{1}H NMR: (400 MHz, $CDCl_3$)

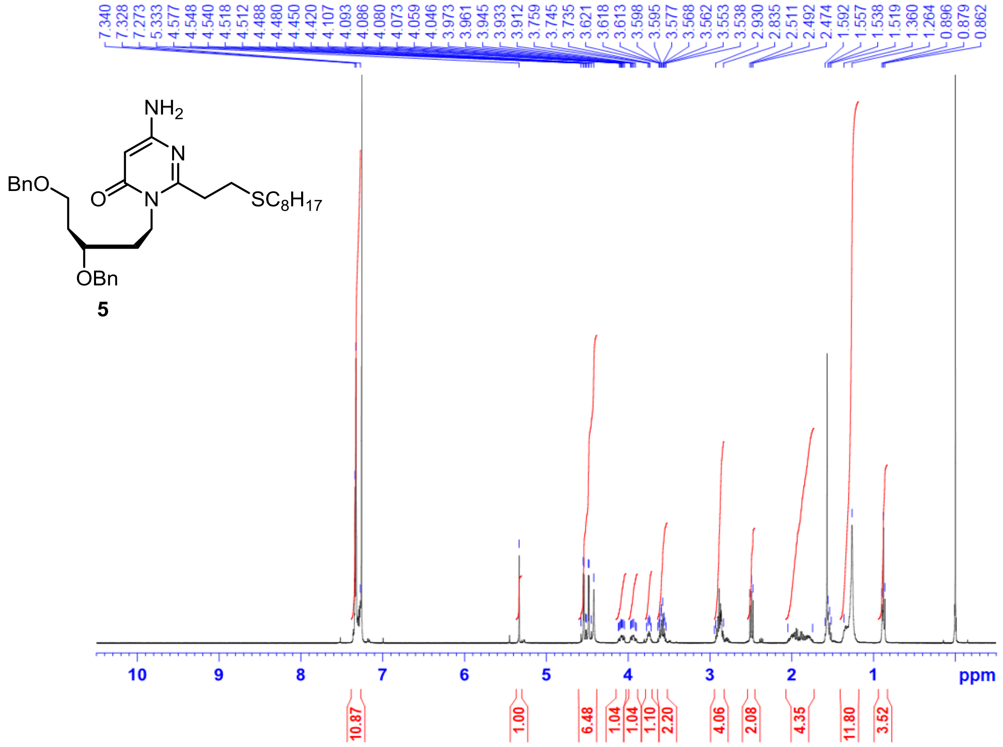

^{13}C NMR: (100 MHz, $CDCl_3$)

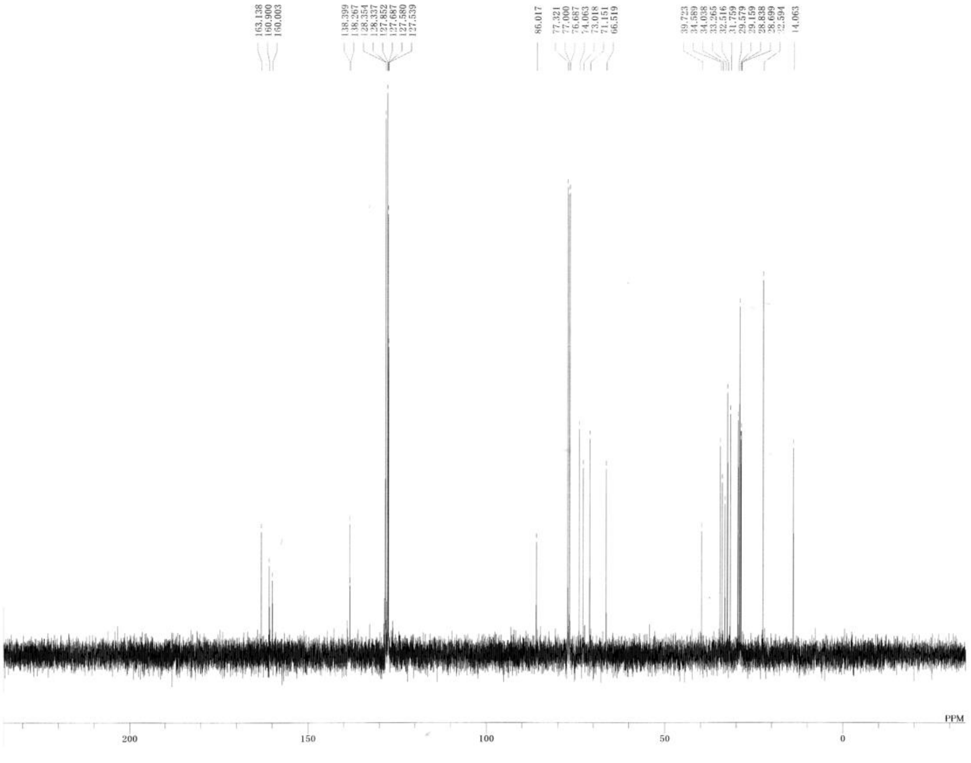

HMBC: (recorded on 600 MHz, $CDCl_3$)

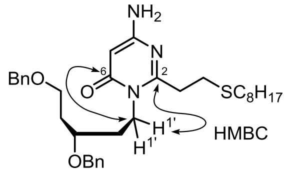

6

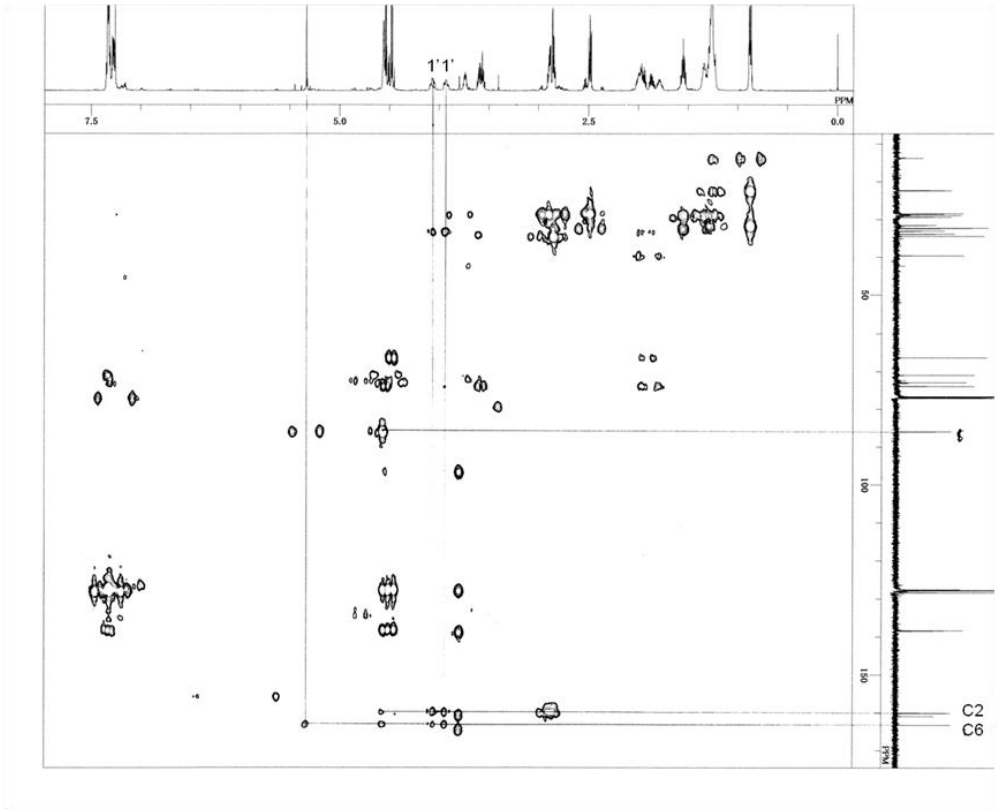

2-(2-octylthioethyl)-6-oxo-4-phenoxyacetylamino-1-(3’,5’-*O*-dibenzyl-2’,4’-dideoxy-D-ribityl)pyrimidine (6)

^{1}H NMR: (400 MHz, $CDCl_3$)

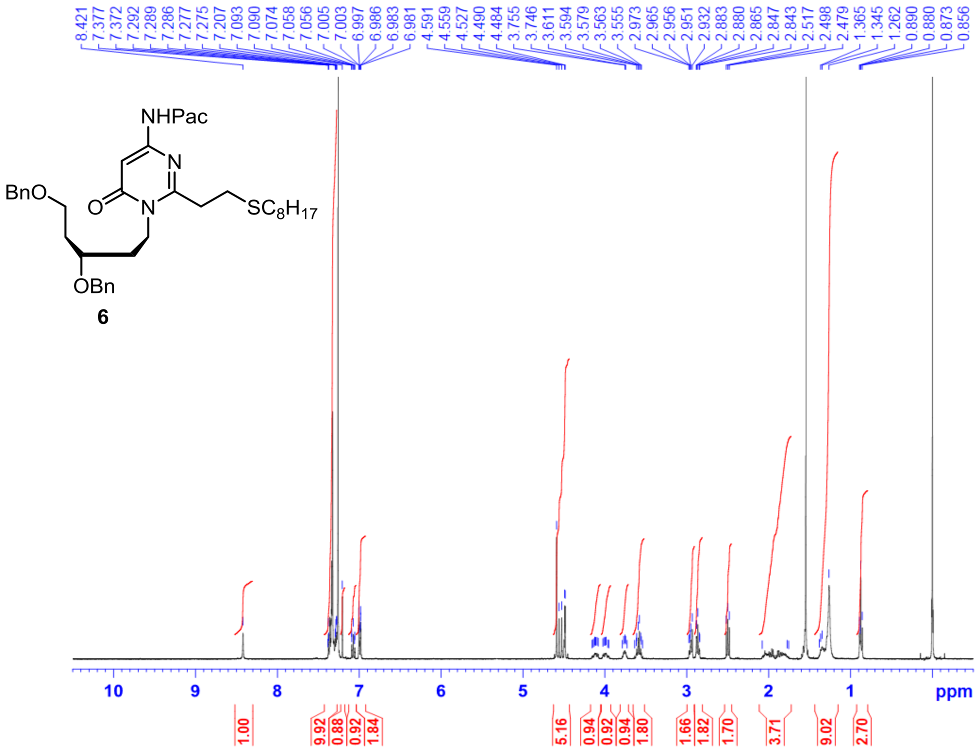

^{13}C NMR: (100 MHz, $CDCl_3$)

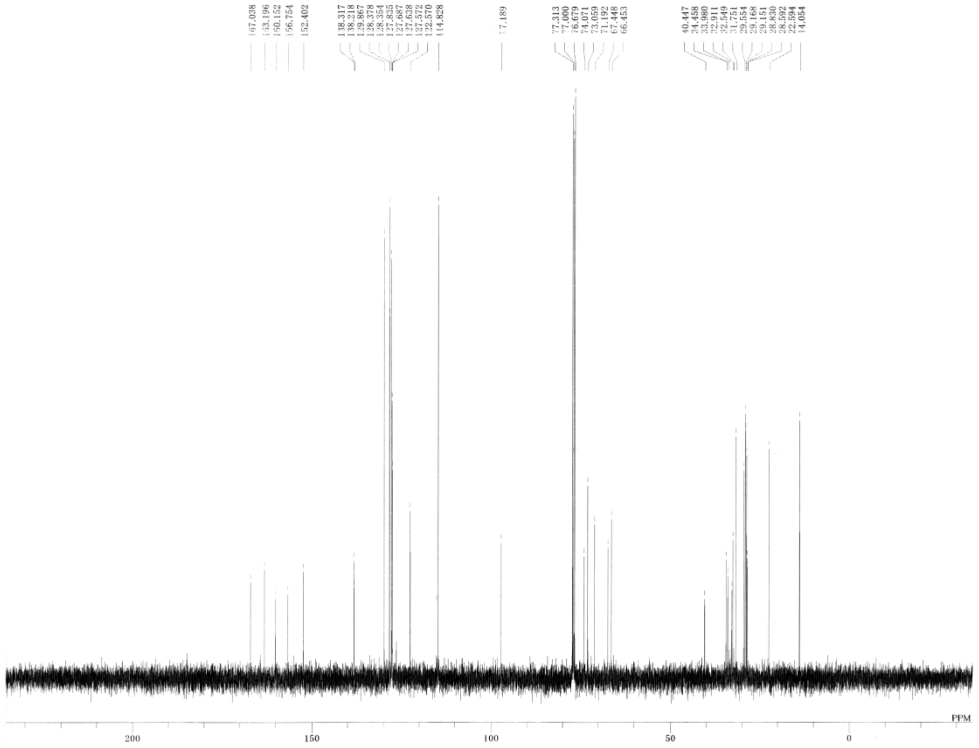

2-(2-octylthioethyl)-6-oxo-4-phenoxyacetylamino-1-(2',4'-dideoxy-D-ribityl)pyrimidine (7)

^{1}H NMR: (400 MHz, $CDCl_3$)

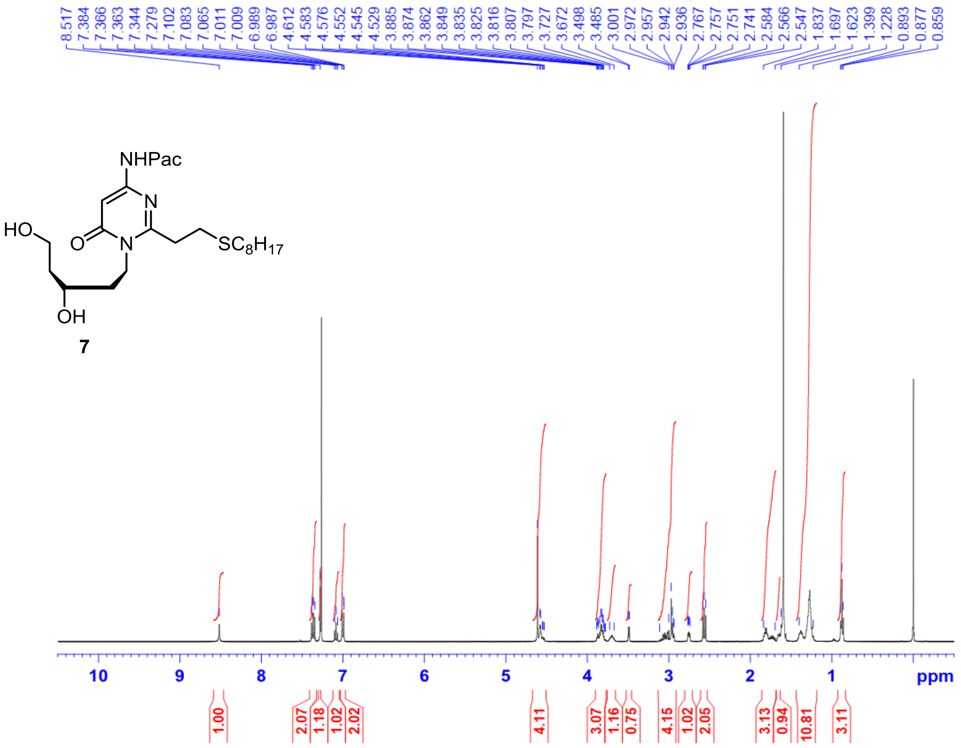

^{13}C NMR: (100 MHz, $CDCl_3$)

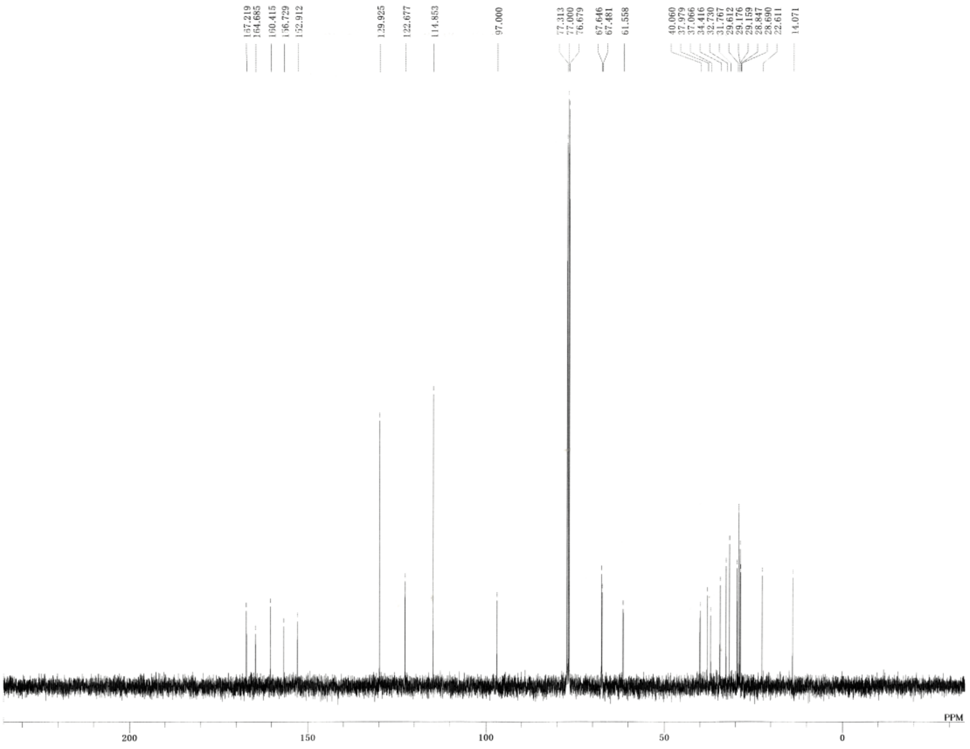

2-(2-octylthioethyl)-6-oxo-4-phenoxyacetylamino-1-(5'-*O*-(4,4'-dimethoxytrityl)-2',4'-dideoxy-D-ribityl)pyrimidine (8)

^{1}H NMR: (400 MHz, $CDCl_3$)

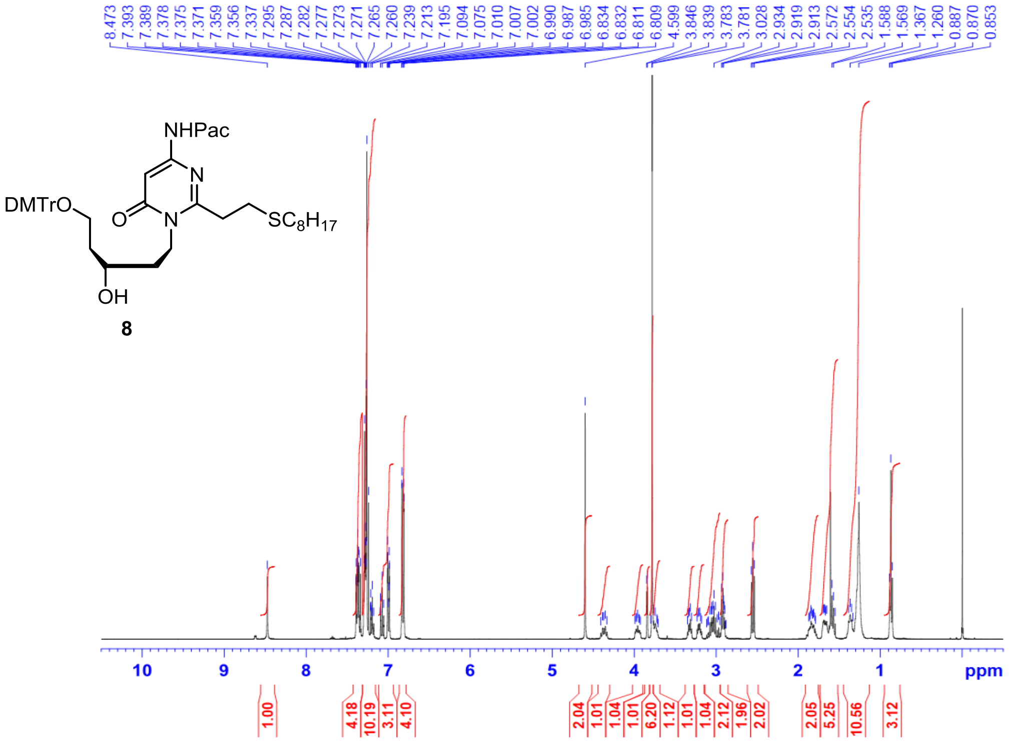

^{13}C NMR: (100 MHz, $CDCl_3$)

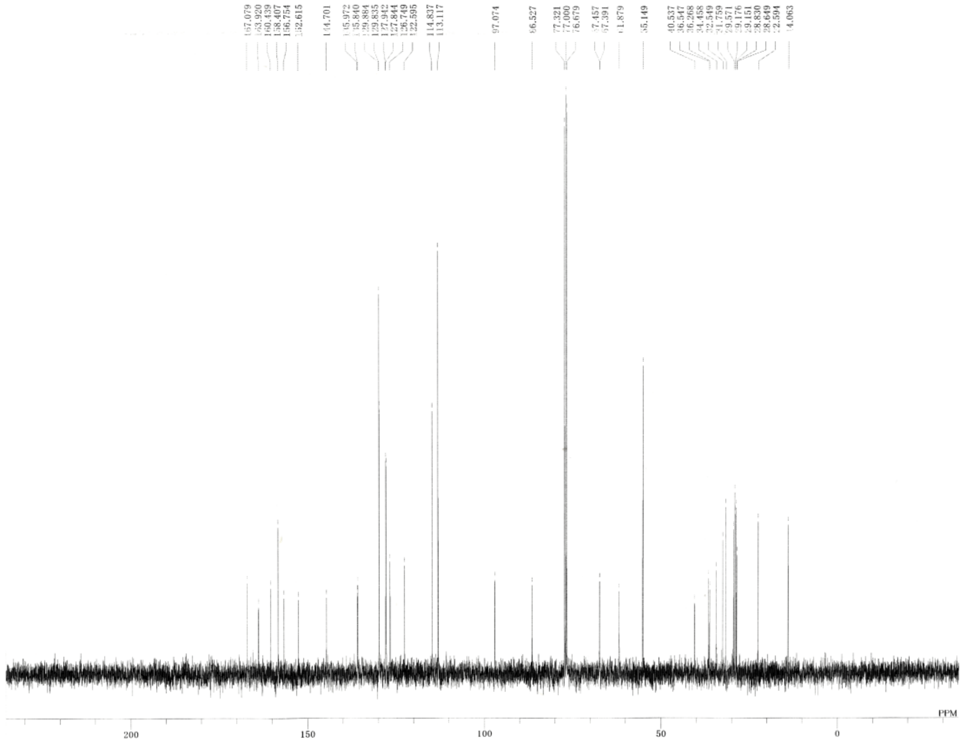

2-(2-octylthioethyl)-6-oxo-4-phenoxyacetylamino-1-(3’-*N*,*N*-diisopropylcyanoethylphosphoramidyl-5’-*O*-(4,4’-dimethoxytrityl)-2’,4’-dideoxy-D-ribityl)pyrimidine (9)

^{1}H NMR: (400 MHz, $CDCl_3$)

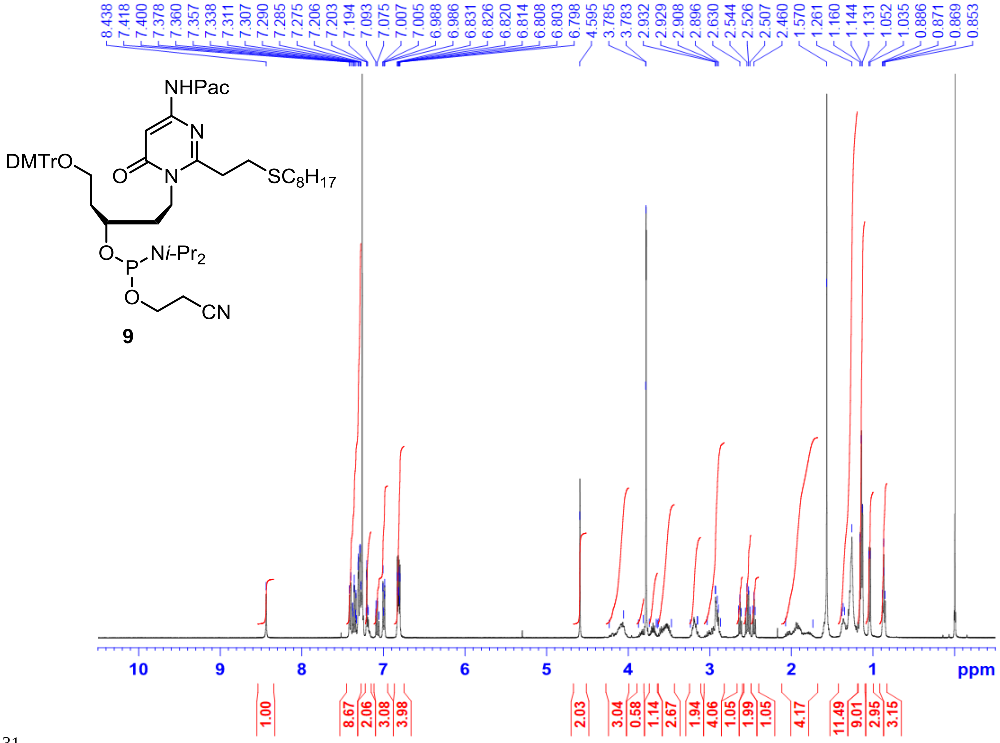

^{31}P NMR:

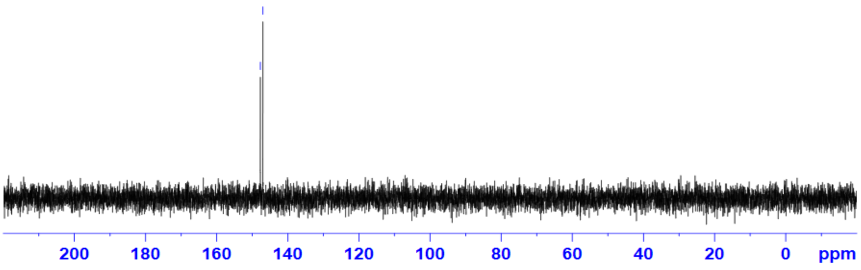

· **Synthesis of oligonucleotide ODN4 bearing 4-amino-2-ethyl-6-oxopyrimidine (AEOP):** To a solution of **ODN3** (47.6 μl, 5 nmol), was added a solution of sodium boron hydride (2.5 μl, 250 nmol) in H_2O at room temperature. After 1 h, the crude product was purified by reverse-phase HPLC with C-18 column (nacalai tesque: COSMOSIL 5C18-MS-II, 4.6 × 250 mm) by a linear gradient of 10-40%/20 min of acetonitrile in 0.1 M TEAA buffer at a flow rate of 1 ml/min to afford ODN**4**; MALDI-TOF MS (*m*/*z*): $[M-H]^-$ calcd 3900.6; found 3899.9.

(A) HPLC profile of **ODN1** after purification

(B) HPLC trace for the synthesis of **ODN3**

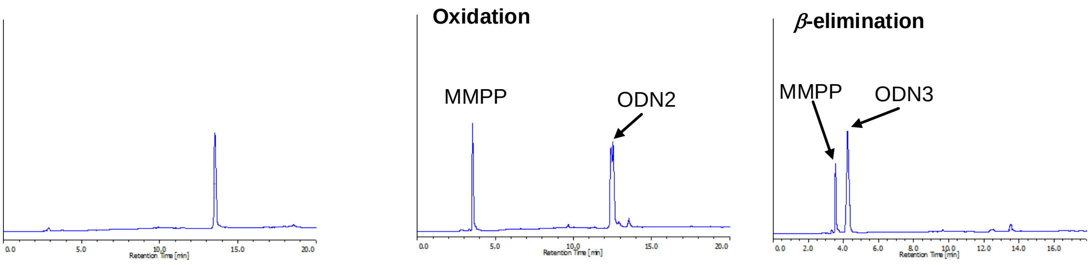

Fig 1S HPLC profile of **ODN1** and **ODN3**

· T_m **analysis:** All samples for T_m experiments were consisted of 100 mM NaCl, 25 mM MES buffer (pH 7) and [Duplex] = 1 μM. T_m experiments were performed with temperature controller. Both the heating and cooling curves were measured over a temperature range of 25 °C to 80 °C at 0.5 °C/min in three times. The absorbance at 260 nm was recorded every 0.5 °C.

Table 1S The melting temperature for the duplex of **ODN4** and **DNA 1**.

ODN4: 5'-d(CCGCGT-**X**-TCGCCG)-3'

DNA1: 3'-d(GGCGCA-**Y**-AGCGGC)-5'

X	Y	Tm (° C)
C	G<br>I<br>8-oxoG<br>2-AP	65.1±0.3<br>59.8±0.3<br>64.4±0.6<br>54.7±0.1
A	G<br>I<br>8-oxoG	54.5±1.1<br>58.8±0.3<br>60.2±0.4
AEOP	G<br>I<br>8-oxoG<br>2-AP	43.8±1.1<br>49.8±0.6<br>48.5±1.2<br>52.5±1.2
T	2-AP	61.0±1.4

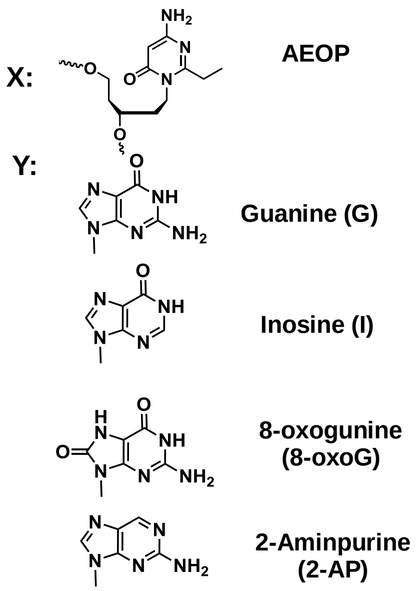

T_m was measured using 1.0 μM each of the strand in 100 mM NaCl, 50 mM MES buffer, pH 7.0.

Crosslinking reactivity with difference ratio of ODN3 to target DNA or RNA.

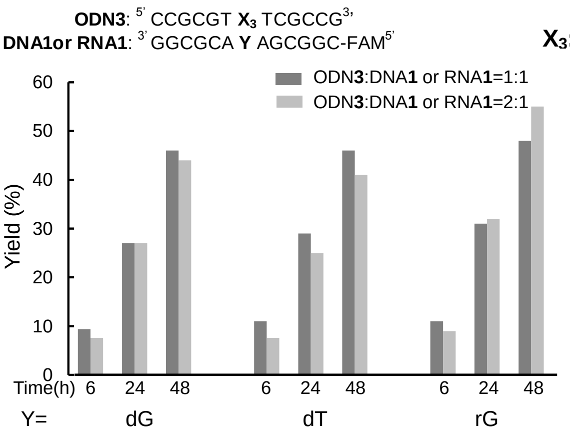

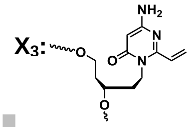

Fig. 2S Crosslinking reactivity with difference ratio of **ODN3** to target **DNA1** or **RNA1**

The reaction was performed using 10 μM **ODN3** and 5 μM **DNA1**, **RNA1** or 10 μM **ODN3** and 10 μM **DNA1**, **RNA1** , in 100 mM NaCl, 50 mM MES, pH 7 at 37 ºC.

There were no significant differences in the crosslink yields when the crosslinking reactions were carried out in 1:1 ratios of ODN**3** versus target DNA or RNA.

- **Hydroxyl radical cleavage**

To a solution of fluoresceine labeled purified adduct (70.7 μM, 4.95 μL, 350 pmol) in H_2O (3.05 μL), were added a solution of 2×oxidation buffer (10 μL, 20 mM NaCl, 20 mM phosphate buffer (pH 7.0), 2 mM sodium ascorbate, 1 mM H_2O_2) and Fe/EDTA solvent (2 μL, 1 mM EDTA, 0.5 mM, $Fe(NH_4)_2(SO_4)_2$). After 3 min and 6 min, the reaction was quenched with 200 mM thio urea (5 μL) and the reaction mixture was dried with speed-vac. The residue was dissolved to 4 μl loading buffer without dyes (95% formamide) and run on a denaturing 20% polyacrylamide gel next to hydoroxy radical cleavage products of target sequence (**DNA1**).

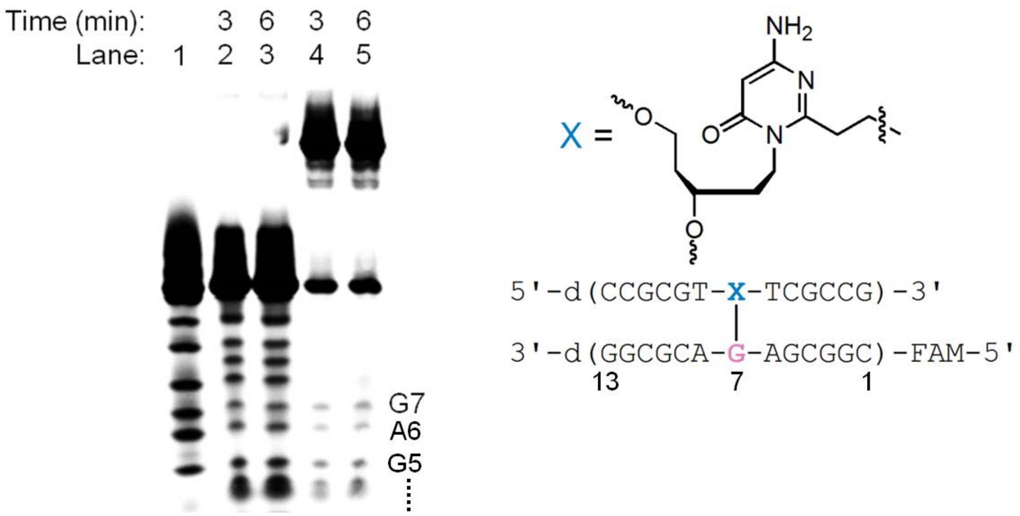

Fig. 3S The hydroxyl radical cleavage of the crosslink adduct of **ODN3** and **DNA1** (**Y** = G). Lane 1: G/A sequencing of **DNA1.** Hydroxy radical cleavage of **DNA1** (Lane 2 and 3) and FAM labeled crosslink adduct (lane 4 and 5). **DNA1** and crosslink adduct were treated with OH· for indicated time.

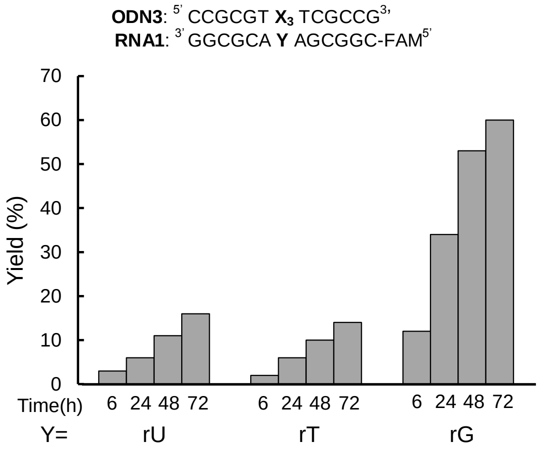

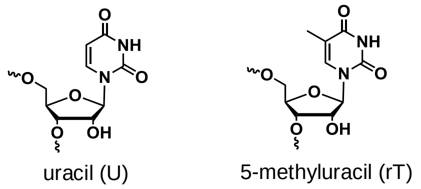

The reaction was performed using 10 μM ODN**3** and 5 μM RNA**1** in 100 mM NaCl, 50 mM MES, pH 7 at 37 ºC.

No significant difference in the crosslink yield was observed for uracil (U) and 5-methyl uracil (rT) in RNA

Fig. 4S Crosslinking reactivity to RNA**1** contained uracil (U), 5-methyluracil (rT), and rG

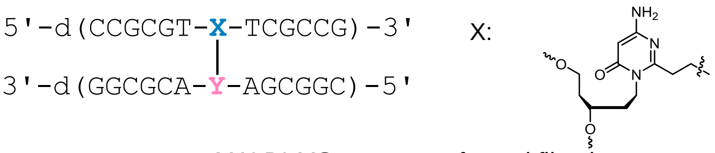

(A) Adduct (**10**) **Y**=Guanine

HPLC profile after gel filtration

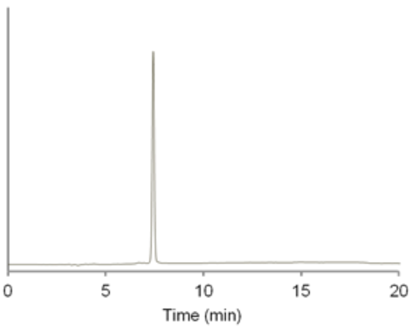

MALDI-MS spectrum after gel filtration

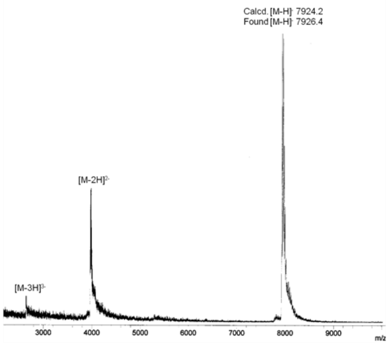

(B) Adduct (**11**) **Y**=8-oxo guanine

HPLC profile after gel filtration

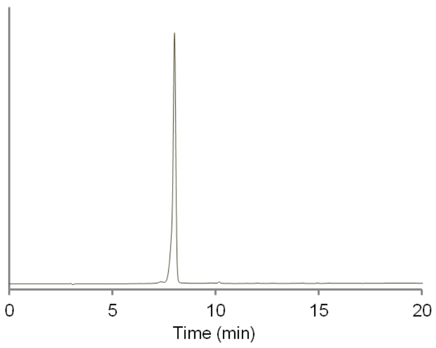

MALDI-MS spectrum after gel filtration

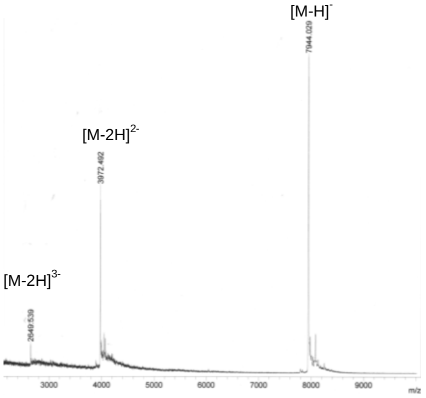

Fig 5S The HPLC profiles and MALDI-MS spectrum after gel filtration of adducts (**10**) and (**11**).

· NMR sample.

A 0.85 mM DNA sample was prepared by dissolving the crosslinked DNA into 267 µL of buffer solution in a Shigemi NMR tube containing 60 mM sodium chloride, 10 mM sodium phosphate (pH 6.5) and 0.01 mM 2,2-dimethyl-2-silapentane-5-sulfonic acid (DSS). For studying the labile proton signals, the buffer solution was made to contain 95% H_2O/5% D_2O. For studying the non-labile signals, the solvent was exchanged to 100% D_2O.

· NMR Experiments

All NMR experiments were performed on two Bruker spectrometers operating at 600.13 and 600.33 MHz, respectively. For studying labile proton signals, water suppression was done by excitation sculpting pulse sequence in both one-dimensional (1D) and two-dimensional (2D) experiments.[1] For studying nonlabile proton signals, residual water signal was suppressed by a 1.4 s presaturation pulse. 2D nuclear Overhauser effect spectroscopy (NOESY) at a mixing time of 180, 200, 250, 300, 350, 400 and 150 ms, double-quantum filtered correlation spectroscopy (DQF-COSY), and total correlation spectroscopy (TOCSY) at a mixing time of 75 and 150 ms were performed. In general, 4096 × 512 data sets were collected for homonulear 2D experiments and the acquired data were zero-filled to give 4096 × 4096 spectra with a 60°-phase-shifted sine squared window function applied to both dimensions. 2D experiments were conducted at various temperatures in the range of 5 to 40 ºC in order to better observe and resolve the labile and non-labile signals.

For carbon resonance assignments, three ^{1}H-^{13}C heteronuclear single quantum correlation (HSQC) experiments,[2-4] focusing on (i) H2/H6/H8-C2/C6/C8, (ii) H5/H1'/H3'-C5/C1'/C3', and (iii) H7-C7 and H2'/H2''-C2' regions, respectively, were performed at 40 °C. The proton spectral width was 11 ppm with the carrier frequency positioned at the residual HDO signal. The ^{13}C spectral widths for the three regions were 40, 40, and 30 ppm and the corresponding carrier frequencies were positioned at 26, 90, and 145 ppm, respectively. Data sets of 4096 × 200, 4096 × 440, and 4096 × 380 were acquired. Heteronuclear decoupling was executed by the GARP-4 sequence.[5] Zero-filling and baseline corrections were applied to both dimensions to generate a 4096 × 4096 data matrix. Carbon chemical shifts were indirectly referenced to DSS using the derived nucleus-specific ratio (Ξ) of 0.251449530.

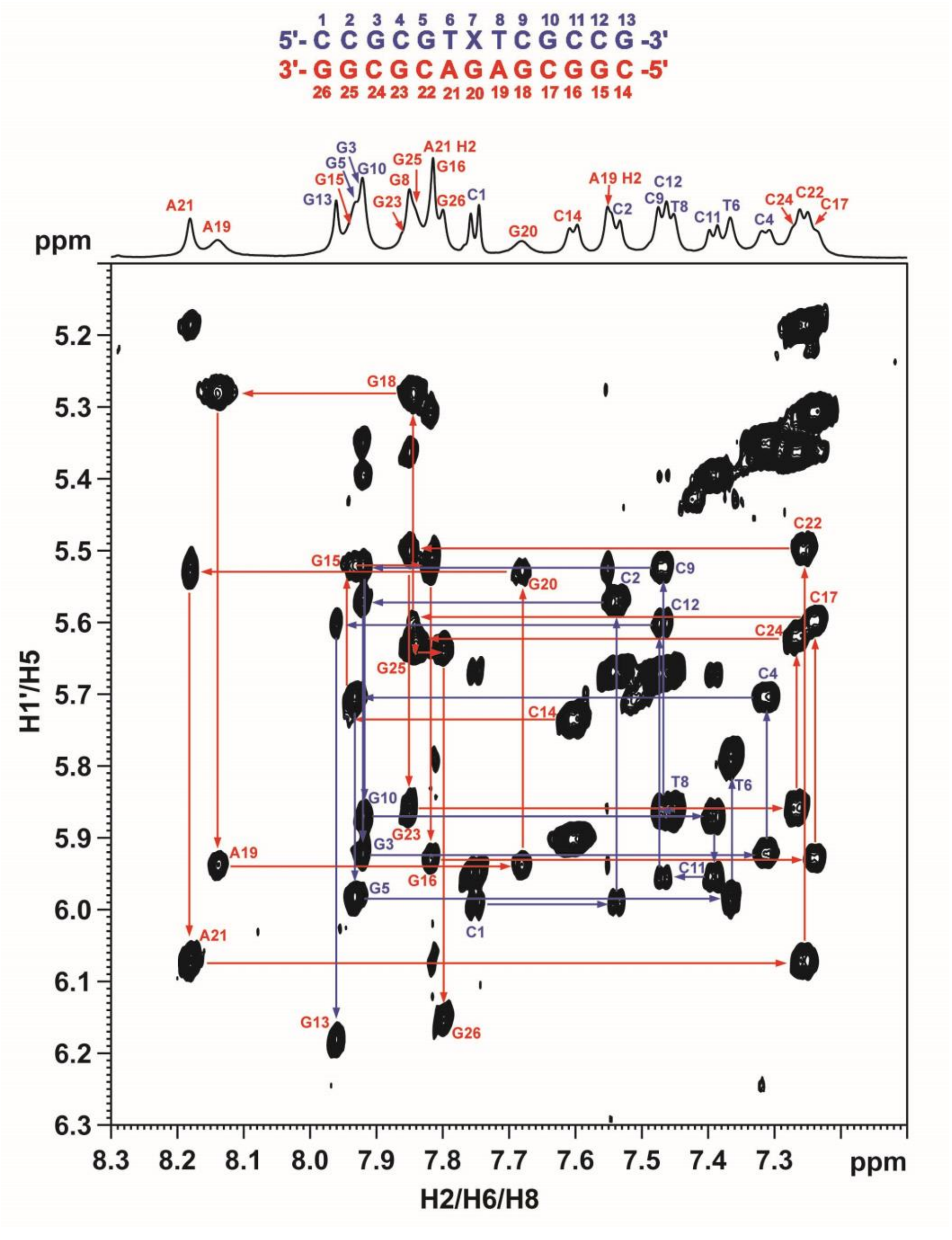

Fig. 6S. Sequential NOE assignment of the crosslink product between ODN3 and DNA1 (Y=G). NOESY was acquired at 20 °C and a mixing time of 300 ms.

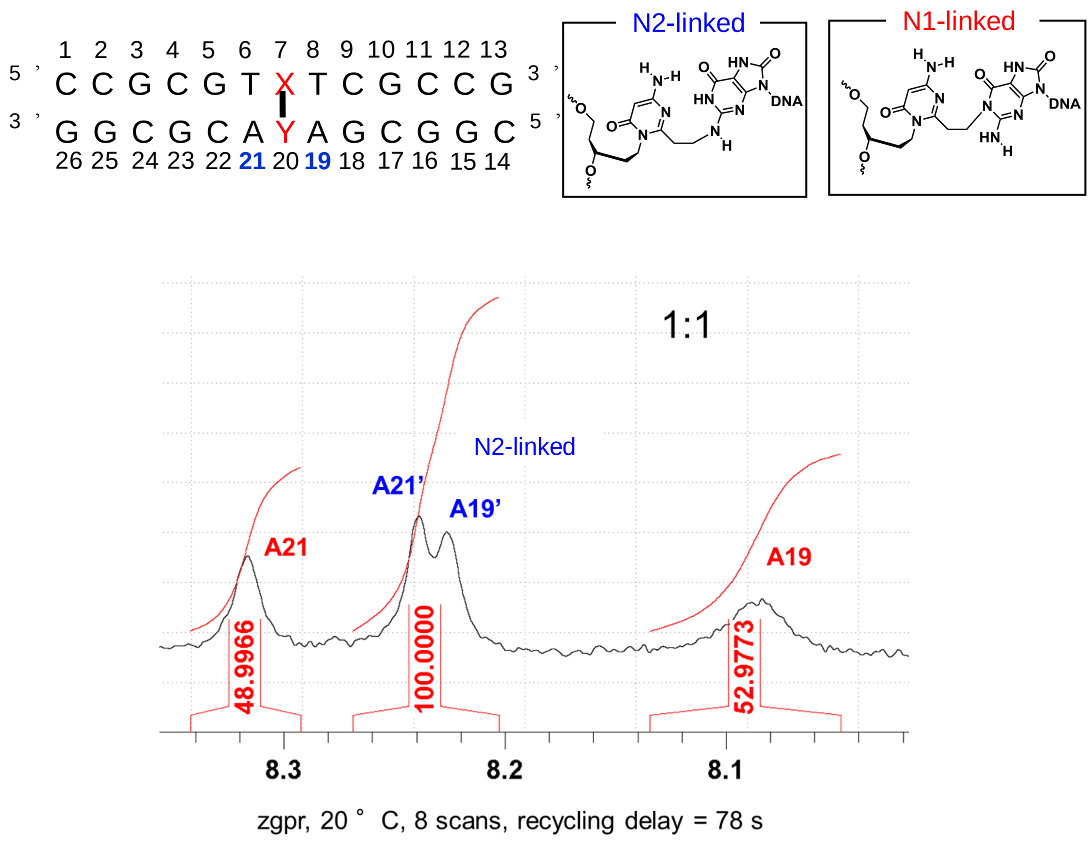

Fig. 7S ^{1}H NMR spectrum with a 78 s recycling delay for accurate peak integral measurements.

· **Computational method**

Geometry optimization and transition state (TS) search calculations were performed, and the obtained geometries were characterized as minimum structures or TS structures on the basis of their harmonic vibrational frequencies and the number of imaginary frequencies. The theoretical level of MP2(full)/6-31G* was used for all the calculations. The polarizable continuum model using integral equation formalism (IEFPCM) was used to consider the hydration effect. All calculations were performed using Gaussian 09 program package.[1] The calculations were carried out at the Center for Quantum Life Sciences (QuL*i*S) and at the Research Center for Computational Science, Okazaki National Research Institutes.

[1] M.J. Frisch, G.W. Trucks, H.B. Schlegel, G.E. Scuseria, M.A. Robb, J.R. Cheeseman, G. Scalmani, V. Barone, B. Mennucci, G.A. Petersson, H. Nakatsuji, M. Caricato, X. Li, H.P. Hratchian, A.F. Izmaylov, J. Bloino, G. Zheng, J.L. Sonnenberg, M. Hada, M. Ehara, K. Toyota, R. Fukuda, J. Hasegawa, M. Ishida, T. Nakajima, Y. Honda, O. Kitao, H. Nakai, T. Vreven, J.A. Montgomery, Jr., J.E. Peralta, F. Ogliaro, M. Bearpark, J.J. Heyd, E. Brothers, K.N. Kudin, V.N. Staroverov, R. Kobayashi, J. Normand, K. Raghavachari, A. Rendell, J.C. Burant, S.S. Iyengar, J. Tomasi, M. Cossi, N. Rega, J.M. Millam, M. Klene, J.E. Knox, J.B. Cross, V. Bakken, C. Adamo, J. Jaramillo, R. Gomperts, R.E. Stratmann, O. Yazyev, A.J. Austin, R. Cammi, C. Pomelli, J.W. Ochterski, R.L. Martin, K. Morokuma, V.G. Zakrzewski, G.A. Voth, P. Salvador, J.J. Dannenberg, S. Dapprich, A.D. Daniels, Ö. Farkas, J.B. Foresman, J.V. Ortiz, J. Cioslowski, D.J. Fox, Gaussian 09, Revision D.01, Gaussian, Inc., Wallingford CT, (2009).
